# The risk of nephrotic range proteinuria and kidney failure in primary laminopathies is genotype specific

**DOI:** 10.1172/jci.insight.192062

**Published:** 2026-07-16

**Authors:** Sebastian Sewerin, Charlotte Aurnhammer, Mohamed Hamed, Gwladys Revêchon, Ria Schönauer, Christin Findeisen, Konstanze Miehle, Šárka Tesařo vá, Theodoros Georgomanolis, Carsten Bergmann, Constantin Wolff, Marek Kollár, Baris Akinci, David Araújo-Vilar, Giovanni Ceccarini, Éva Csajbók, Alessandra Gambineri, Martin Heni, Thomas Scherer, Iztok Štotl, Ekaterina Sorkina, Marie-Christine Vantyghem, Elena Vorona, Martin Wabitsch, Julia von Schnurbein, Camille Vatier, Joëlle Roume, Yves Reznik, Maria Eriksson, Wolfram Antonin, Corinne Vigouroux, Jan Halbritter

**Affiliations:** 1 Division of Nephrology, University of Leipzig Medical Center, Leipzig, Germany.; 2 Broad Institute of MIT and Harvard, Cambridge, Massachusetts, USA.; 3 III. Department of Medicine, University Medical Center Hamburg-Eppendorf, Hamburg, Germany.; 4 Hamburg Center for Kidney Health (HCKH), University Medical Center Hamburg-Eppendorf, Hamburg, Germany.; 5 Division of Hepatology, University of Leipzig Medical Center, Leipzig, Germany.; 6 Institute of Biochemistry and Molecular Cell Biology, Medical School, RWTH Aachen University, Aachen, Germany.; 7 Department of Medicine Huddinge, Karolinska Institute, Stockholm, Sweden.; 8 Department of Nephrology and Medical Intensive Care, Charité Universitätsmedizin Berlin, Berlin, Germany.; 9 Division of Endocrinology, Lipodystrophy Center Leipzig, University of Leipzig Medical Center, Leipzig, Germany.; 10 3rd Department of Internal Medicine, 1st Faculty of Medicine, Charles University and General University Hospital, Prague, Czech Republic.; 11 Cologne Center for Genomics, University of Cologne, Cologne, Germany.; 12 Medizinische Genetik Mainz, Limbach Genetics, Mainz, Germany.; 13 Institute for Clinical and Experimental Medicine, Prague, Czech Republic.; 14 DEPARK, Dokuz Eylul University Health Campus and Izmir Biomedicine and Genome Center, Izmir, Turkey.; 15 Centre for Research in Molecular Medicine and Chronic Diseases (CiMUS), University of Santiago de Compostela, Santiago de Compostela, Spain.; 16 Obesity and Lipodystrophy Center, Department of Clinical and Experimental Medicine, Endocrinology Unit, University Hospital of Pisa, Pisa, Italy.; 17 University of Szeged, Faculty of Medicine, Szent-Györgyi Albert Clinical Center, 1st Department of Internal Medicine, Endocrinology Department, Szeged, Hungary.; 18 Department of Medical and Surgical Sciences, University of Bologna, Bologna, Italy.; 19 Division of Endocrinology and Diabetology, Department of Internal Medicine I, Ulm University Hospital, Ulm, Germany.; 20 Department for Diagnostic Laboratory Medicine, Institute for Clinical Chemistry and Pathobiochemistry, Eberhard Karls University Tübingen, Tübingen, Germany.; 21 Division of Endocrinology and Metabolism, Department of Medicine III, Medical University of Vienna, Vienna, Austria.; 22 Department of Endocrinology, Diabetes and Metabolic Diseases, University Medical Centre Ljubljana, Ljubljana, Slovenia.; 23 Endocrinology Research Centre, Moscow, Russia.; 24 Department of Endocrinology and Metabolism, Inserm U 1190, Lille University Hospital, Lille, France.; 25 Division of Endocrinology, Diabetology and Nutritional Medicine, Department of Medicine B: Gastroenterology, Hepatology, Endocrinology and Clinical Infectiology, University Hospital Münster, Münster, Germany.; 26 Center for Rare Endocrine Diseases, Division of Pediatric Endocrinology and Diabetes, Department of Pediatrics and Adolescent Medicine, Ulm University Hospital, Ulm, Germany.; 27 Assistance Publique-Hôpitaux de Paris, Saint-Antoine Hospital, Department of Endocrinology, Diabetology, and Reproductive Endocrinology, National Reference Center for Rare Diseases of Insulin Secretion and Insulin Sensitivity (PRISIS), Paris, France.; 28 Sorbonne University, Inserm UMR 938, Research Center Saint-Antoine, Paris, France.; 29 Foundation for Innovation in Cardiometabolism and Nutrition (IHU ICAN), Paris, France.; 30 Department of Genetics, Poissy-Saint-Germain-en-Laye Medical Center, Poissy and Saint-Germain-en-Laye, France.; 31 Department of Endocrinology and Diabetes, University of Caen Medical Center Côte de Nacre, and University of Caen Normandy, Caen, France.; 32 Renal Division, Department of Medicine, Faculty of Medicine and Medical Center - University of Freiburg, Freiburg, Germany.

**Keywords:** Genetics, Nephrology, Chronic kidney disease

## Abstract

**BACKGROUND:**

Primary laminopathies are a heterogeneous group of rare diseases caused by nuclear lamina dysfunction due to pathogenic *LMNA* variants. However, despite their ubiquitous expression, *LMNA* variants have rarely been linked to chronic kidney disease (CKD). Here, we systematically investigate clinical implications and functional underpinnings of a distinct *LMNA* missense variant [lamin A/C p.(Arg349Trp)] that has sporadically been found in patients with a complex phenotype including lipodystrophy, proteinuria, and focal segmental glomerulosclerosis (FSGS).

**METHODS:**

In clinical and functional terms, we compare lamin A/C Arg349Trp with missense changes at Arg482, the most common hotspot residue for type 2 familial partial lipodystrophy (FPLD2). In particular, we assess renal endpoints in corresponding patient cohorts and investigate disease-associated alterations in vitro.

**RESULTS:**

In contrast to patients with FPLD2, individuals with lamin A/C Arg349Trp experience high-grade proteinuria and a rapid decline of glomerular filtration rate with kidney failure at a median age of 43 years. Mechanistically, we demonstrate that Arg349Trp associates with an abrogation of the structural interaction between lamin A/C and nucleoporin 155, nuclear pore complex aggregation, and an alteration of TGF-β1–dependent signaling.

**CONCLUSION:**

While patients with Lamin A/C Arg482 missense changes are at very low risk for progressive CKD, patients harboring Arg349Trp show nephrotic range proteinuria and kidney failure in midlife. Hence, high-grade proteinuric kidney disease is genotype specific, and patients with the Arg349Trp substitution require early renoprotective intervention to potentially halt progression and prevent kidney failure.

**FUNDING:**

German Research Foundation, project IDs 502928386, 508310822, 539950728, 445703531, 451693578, 471294925, 496611648, and 539950728; Else Kröner-Fresenius Foundation (2019_A96).

## Abstract 2

Through comparative clinical analyses and cell-based experimentation, a specific *LMNA* variant is shown to be pathogenic and associated with nephrotic range proteinuria and kidney failure.

## Introduction

Well over 400 different mutations in the *LMNA* gene have been reported to date (1), giving rise to a number of rare diseases that define the group of primary laminopathies. Their phenotypic spectrum reaches from rather isolated organ dysfunctions such as dilated cardiomyopathy (MIM# 115200) to multifaceted syndromes such as familial partial lipodystrophy (FPLD, type 2 MIM# 151660) or Hutchinson-Gilford progeria syndrome (HGPS, MIM# 176670), a disorder with premature aging and drastically reduced life expectancy (2). Major *LMNA* gene products are lamins A and C, which are integral components of the nuclear lamina, a filamentous meshwork beneath the inner nuclear membrane. The nuclear lamina is of structural and functional importance, stabilizing the nuclear architecture as well as regulating chromatin organization and gene expression.

Despite evidence of nondiabetic proteinuric kidney disease in laminopathies (3–5), no systematic analysis to study this phenotypic trait has been conducted, to date. While single *LMNA* missense variants [c.898G > C, p.(Asp300His) and c.29C>T, p.(Thr10Ile); NM_170707.4] have occasionally been associated with proteinuria and focal segmental glomerulosclerosis (FSGS) (6), only the variant c.1045C>T, p.(Arg349Trp) (NM_170707.4) has repeatedly and independently been reported in this context (4, 5, 7, 8). Data published on this variant indicate an otherwise variable phenotype with multiorgan involvement, including the heart, skeletal muscle, and adipose tissue (4, 5, 7–11).

The nonspecific histopathological pattern of FSGS reflects podocyte dysfunction, injury, or loss caused by several different etiologies, both environmental and genetic in nature (12). Despite considerable efforts, diagnosis and management of clinical FSGS syndromes remain challenging, and oftentimes, treatment does not prevent kidney failure. Throughout the past 2 decades, much progress has been made in the identification of monogenic causes of podocytopathies. In particular, loss-of-function variants in multiple nuclear pore genes were shown to cause FSGS (13–15). The nuclear lamina, in turn, is closely associated with nuclear pore complexes (NPCs), which are large protein assemblies that constitute selective gateways to the nucleus (16–18). Given the pathogenic link between aberrant nucleoporins and FSGS, we hypothesized that the Arg349Trp substitution in lamin A/C compromises its interaction with NPC components, leading to podocyte dysfunction in a similar way as mutant nucleoporins.

Based on data from a large cohort of patients with type 2 FPLD (FPLD2) due to distinct *LMNA* mutations, we here demonstrate the genuinely nephropathogenic nature of lamin A/C Arg349Trp. In terms of its functional implications on the cellular level, we show that the substitution Arg349Trp impedes TGF-β1–dependent signaling as well as the structural association between lamin A/C and nucleoporin 155, thus indicating a specific mode of podocyte dysfunction that is shared with mutant nucleoporins.

## Results

### Patients with lamin A/C Arg349Trp present with progeria, proteinuric kidney disease, and FSGS.

**Figure 1:**
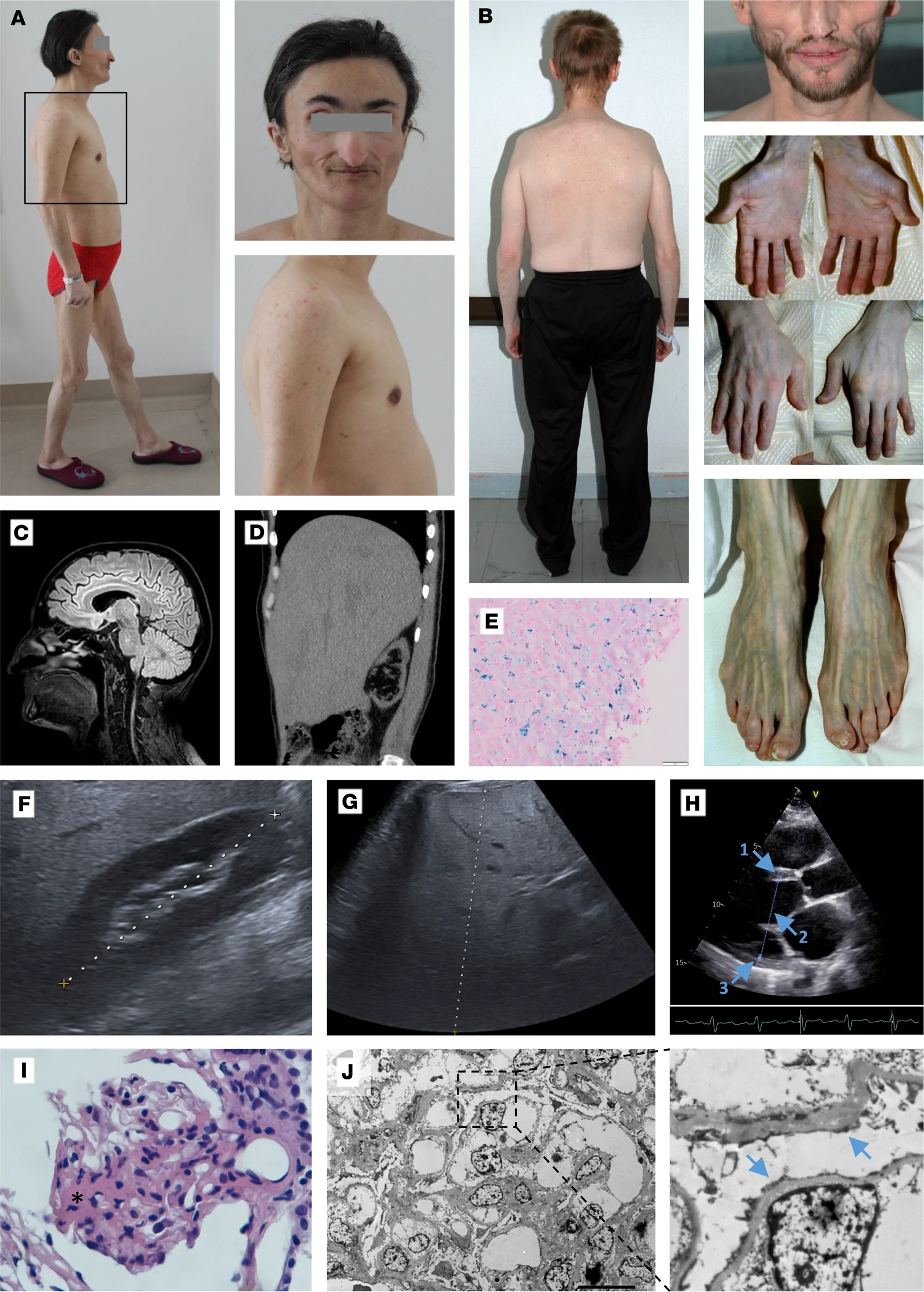
Patients harboring lamin A/C Arg349Trp present with atypical progeroid syndrome and kidney disease. (**A**) Full body picture (left) of an affected female (patient 3.3.1) displaying generalized lipodystrophy as well as progeroid features. Note the missing mammae development (bottom right, close-up of boxed region). (**B**) Full body picture (left) of an affected male (patient 3.10.2) with partial lipodystrophy and progeroid features. Note the paucity of s.c. adipose tissue in face, hands, and feet (right). (**C**–**E**) Clinical findings from the patient shown in **A**. (**C**) Sagittal section of cranial magnetic resonance tomography highlighting generalized cerebral atrophy. (**D**) Sagittal section of computed tomography showing hepatomegaly and a small involuted right kidney. (**E**) Liver biopsy specimen with Elastica van Gieson, Iron, and PAS staining revealing kupffercell siderosis. Scale bar: 100 μm. (**F**–**J**) Clinical findings from another affected female (patient 3.11.1). Ultrasonic images from the right kidney (**F**; dashed line, 113.2 mm) and the liver (**G**; dashed line, 155.8 mm) acquired when the patient was 26 years of age. Note hepatic steatosis and hepato(spleno)megaly. (**H**) Transthoracic echocardiographic image from the same patient at 27 years of age. Parasternal long axis view, highlighting left ventricular and atrial dilations as hallmarks of dilated cardiomyopathy. 1 – interventricular septal thickness, 9 mm (↔); 2 – left ventricular end diastolic diameter, 57 mm (↑); 3 – left ventricular wall thickness, 7 mm (↔). (**I** and **J**) Images from kidney biopsy compatible with FSGS. The biopsy was taken when the patient was 28 years old. (**I**) Glomerulus with segmental sclerosis (asterisk), H&E stain. (**J**) Electron micrograph, showcasing ≥ 80% podocyte foot process effacement (arrows) or podocyte loss.

We identified the heterozygous *LMNA* variant c.1045C>T (NM_170707.4), p.(Arg349Trp), in 4 individuals from 3 independent families. All 4 individuals presented with atypical progeroid syndrome, including lipodystrophy, adult-onset progeria, and insulin resistance with diabetes mellitus in 2 of them (Figure 1, A–H, and Table 1). Additionally, 2 of the 4 individuals developed kidney failure of unknown etiology at age 39 and 40 years, respectively (Table 1). While the other 2 patients were still in their twenties, both developed progressive proteinuria; 1 of them underwent a kidney biopsy, showing glomerular lesions compatible with FSGS (Figure 1, I and J, and Table 1).

With only 2 entries in the population database gnomad (v4.0.0: minor allele frequency 0.0003%), the c.1045C>T variant in *LMNA* is considered ultrarare. Conversely, it has been reported multiple times as a (likely) pathogenic variant in ClinVar (Variation ID: 66762). Given the uncertainty around *LMNA* as a gene associated with genuine kidney disease and the variant’s reported association with proteinuria, we aimed to systematically investigate whether kidney involvement constitutes an obligatory, variable, or no universal trait in this distinct laminopathy. Through systematic literature review (4, 5, 7–11, 19–21) supplemented by direct correspondence with the authors from Izmir, Turkey (20); Philadelphia, Pennsylvania, USA (21); and Pisa, Italy (8), we found 21 individuals with the c.1045C>T, p.(Arg349Trp) *LMNA* variant, of whom 17 had developed proteinuric kidney disease. Of these, 9 were reported to have undergone a kidney biopsy showing FSGS in 7 cases (4, 5, 7, 8, 11, 19).

To expand the scope of available patient data, we next queried the registry curated by the European Consortium of Lipodystrophies (ECLip) for reports of additional cases and their phenotypic characteristics (22). The registry query yielded 2 unpublished cases with lamin A/C Arg349Trp and proteinuric kidney disease (Table 1). Of these, 1 patient had developed kidney failure due to FSGS at age 43.

### Lamin A/C Arg349Trp associates with high levels of proteinuria and kidney failure.

**Figure 2:**
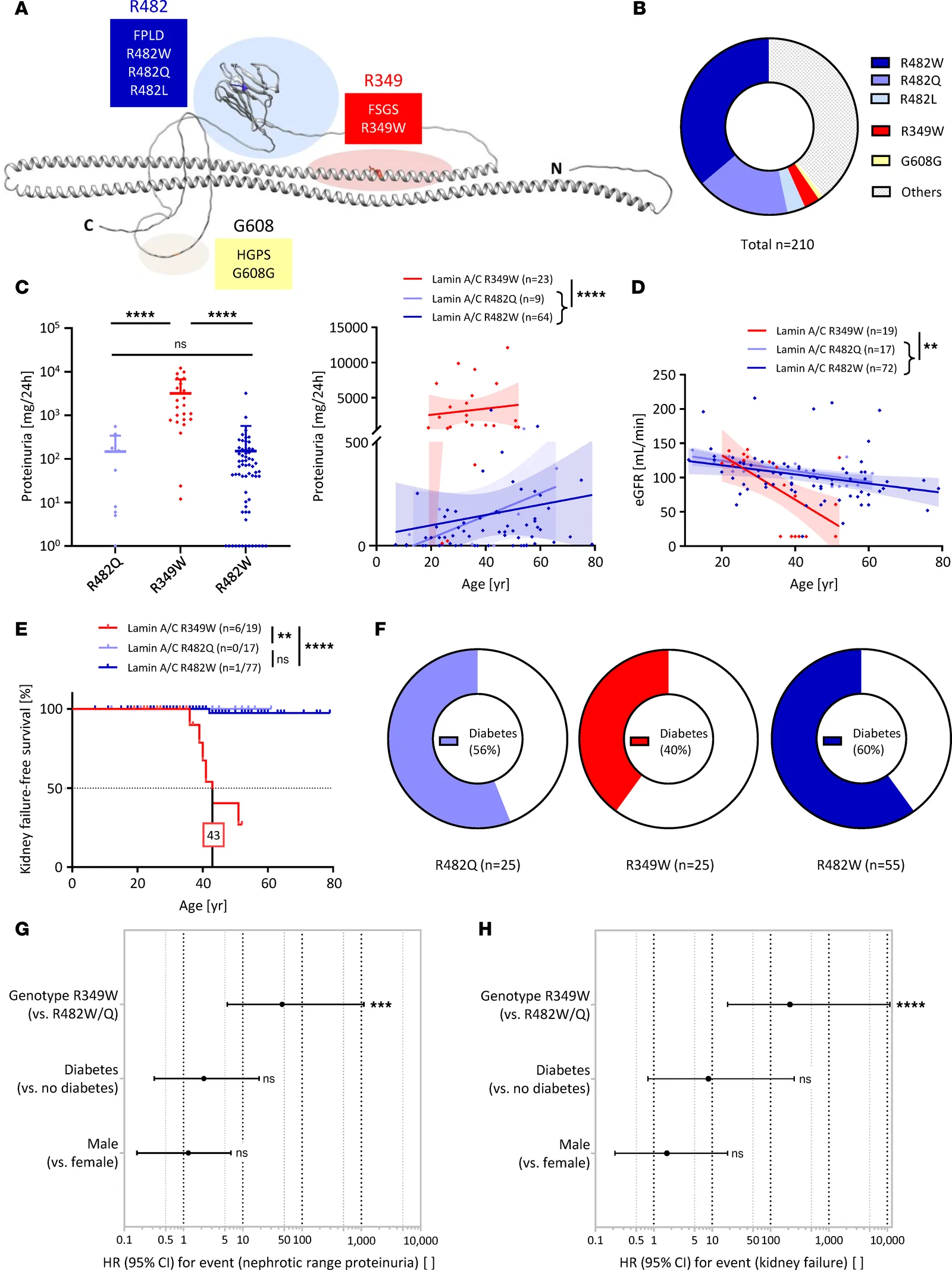
Lamin A/C Arg349Trp associates with high levels of proteinuria and kidney failure. (**A**) AlphaFold modeling of WT lamin A. (**B**) Proportions of patients harboring the indicated lamin A/C protein variants in the entire cohort of patients with primary laminopathies reported through the European Consortium of Lipodystrophies (ECLip) registry. (**C**) With a more than 10-fold difference, proteinuria (mg/24h) was significantly higher in lamin A/C Arg349Trp carriers (red; *n* = 23, mean 3186 mg/24h) as compared with patients harboring the Arg482Trp (dark blue; *n* = 63, mean 151.1 mg/24h) or the Arg482Gln (light blue; *n* = 9, mean 147.9 mg/24h) substitution (left; 1-way ANOVA with Bonferroni-corrected post hoc tests; mean + SD). Pearson correlation between proteinuria and age was not significant for any of the 3 patient cohorts (right). The *y* intercept, but not the slope, of the straight interpolation line for the lamin A/C Arg349Trp cohort differed significantly from that of the joint FPLD cohort (i.e., Arg482Trp or Arg482Gln carriers). The 95% CIs of interpolation lines are indicated by colored bands. For correlations and the interpolation comparison, *P* values were Bonferroni-corrected. Of note, for *n* = 26 individuals only “mg/g creatinine” was available. All groups were tested for the presence of outliers using Grubb’s test, yielding 1 data point in the Arg482Trp group that was excluded from analyses but displayed in the graphs. (**D**) In comparison with the joint cohort of patients harboring either lamin A/C Arg482Trp (dark blue) or Arg482Gln (light blue), the eGFR slope was significantly reduced for the cohort of lamin A/C Arg349Trp patients (red). (**E**) Kaplan-Meier analysis showing a significant reduction in kidney failure-free survival for patients harboring lamin A/C Arg349Trp (red; 31.6% kidney failure at a median age of 43) when compared with those with the Arg482Trp (dark blue; 1.3% kidney failure) or those with the Arg482Gln variant (light blue; 0.0% kidney failure). Log-rank tests with Bonferroni-correction. (**F**) Prevalence of diabetes in cohorts of patients harboring either lamin A/C Arg349Trp or Arg482Trp or Arg482Gln. (**G** and **H**) In comparing lamin A/C Arg349Trp harboring patients with those carrying the Arg482Trp or the Arg482Gln variant, multivariate Cox regression analyses showed that the *LMNA* genotype was a significant covariate for both nephrotic range proteinuria and kidney failure. Median with range. ns, not significant; **P < 0.01; ***P < 0.001; ****P < 0.0001.

To allow for comparative clinical endpoint analyses, we analyzed patient data from the ECLip registry for occurrence of pathogenic variants affecting the most prevalent mutational hotspot (Arg482; Figure 2A) associated with Dunnigan-type FPLD (FPLD2, MIM# 151660): Missense changes at the amino acid residue Arg482 accounted for more than 50% of all patients with *LMNA*-related lipodystrophies in the ECLip registry with 36.2% (*n* = 76/210) for Arg482Trp, 17.1% (*n* = 36/210) for Arg482Gln, and 3.3% (*n* = 7/210) for Arg482Leu (Figure 2B). We thus focused on patients with Arg482Trp or Arg482Gln as groups representative of FPLD2. Upon curation of 2 cohorts of patients with these mutations, we compared their renal phenotypes with those of the group of 27 patients harboring the Arg349Trp substitution who had either been previously reported (*n* = 21) or were identified in this study (*n* = 6) (Figure 2, C–F). For these curations, lamin A/C Arg482Trp patients (*n* = 77) were identified through the ECLip registry, the Lipodystrophy Center Leipzig, Dokuz Eylul University, or the Obesity and Lipodystrophy Center at the University Hospital of Pisa, and patients harboring lamin A/C Arg482Gln (*n* = 36) were identified through the ECLip registry. High-grade proteinuria was almost exclusively associated with lamin A/C Arg349Trp with values ranging about 10-fold above mean proteinuria levels reported for patients with FPLD2 (Figure 2C). The age-related eGFR decline was significantly more pronounced for the cohort of Arg349Trp carriers when compared with the cohort of patients harboring either lamin A/C Arg482Trp or Arg482Gln (Figure 2D). Most strikingly, kidney failure was essentially restricted to patients with lamin A/C Arg349Trp. While 31.6% (*n* = 6/19) of all Arg349Trp carriers exhibited kidney failure at a median age of 43 years, only a single patient (1.3%; *n* = 1/77) with the Arg482Trp substitution was affected by kidney failure and none of the Arg482Gln carriers (*n* = 0/17) (Figure 2E). Of note, the frequency of diabetes mellitus was lower in the cohort of patients with lamin A/C Arg349Trp than in the patient cohorts defined by the Arg482Trp or Arg482Gln substitutions (Figure 2F). From the covariates *LMNA* genotype, occurrence of diabetes mellitus, and sex, only the presence of lamin A/C Arg349Trp significantly associated with both nephrotic range proteinuria and kidney failure (Figure 2, G and H).

### Lamin A/C Arg349Trp associates with nuclear pore complex aggregation.

In patients with progeroid syndromes caused by different heterozygous *LMNA* mutations, nuclear shape abnormalities have been found in dermal fibroblasts to an increased yet varying extent (7, 23–25). To compare nuclear morphology between cells expressing lamin A/C WT, Arg349Trp, Arg482Trp, and Gly608Gly, nuclear shapes were studied in standardized cell models following immunofluorescence labeling of lamin A/C. Note that the synonymous mutation c.1824C>T, which underlies Gly608Gly and causes HGPS, activates a cryptic donor splice site, resulting in an aberrant lamin A precursor protein, the so-called progerin, which is not processed as its WT counterpart is (23, 24).

Upon transient transfection of immortalized human podocytes (26) with WT and mutant *LMNA* constructs, exogenous proteins localized to the nuclear envelope (Supplemental Figure 1A; supplemental material available online with this article; https://doi.org/10.1172/jci.insight.192062DS1). There was no significant difference in the amount of abnormally shaped nuclei between genotypes (Supplemental Figure 1B). This held true for both double (i.e., WT plus individual mutants) and single transfections. Similar results were obtained from transiently transfected HEK293T cells (Supplemental Figure 1, C and D). In these assays, morphological data were acquired by 2 observers blinded to the experimental conditions and analyzed separately, yielding similar results. These findings are consistent with results from a previous report (7).

**Figure 3:**
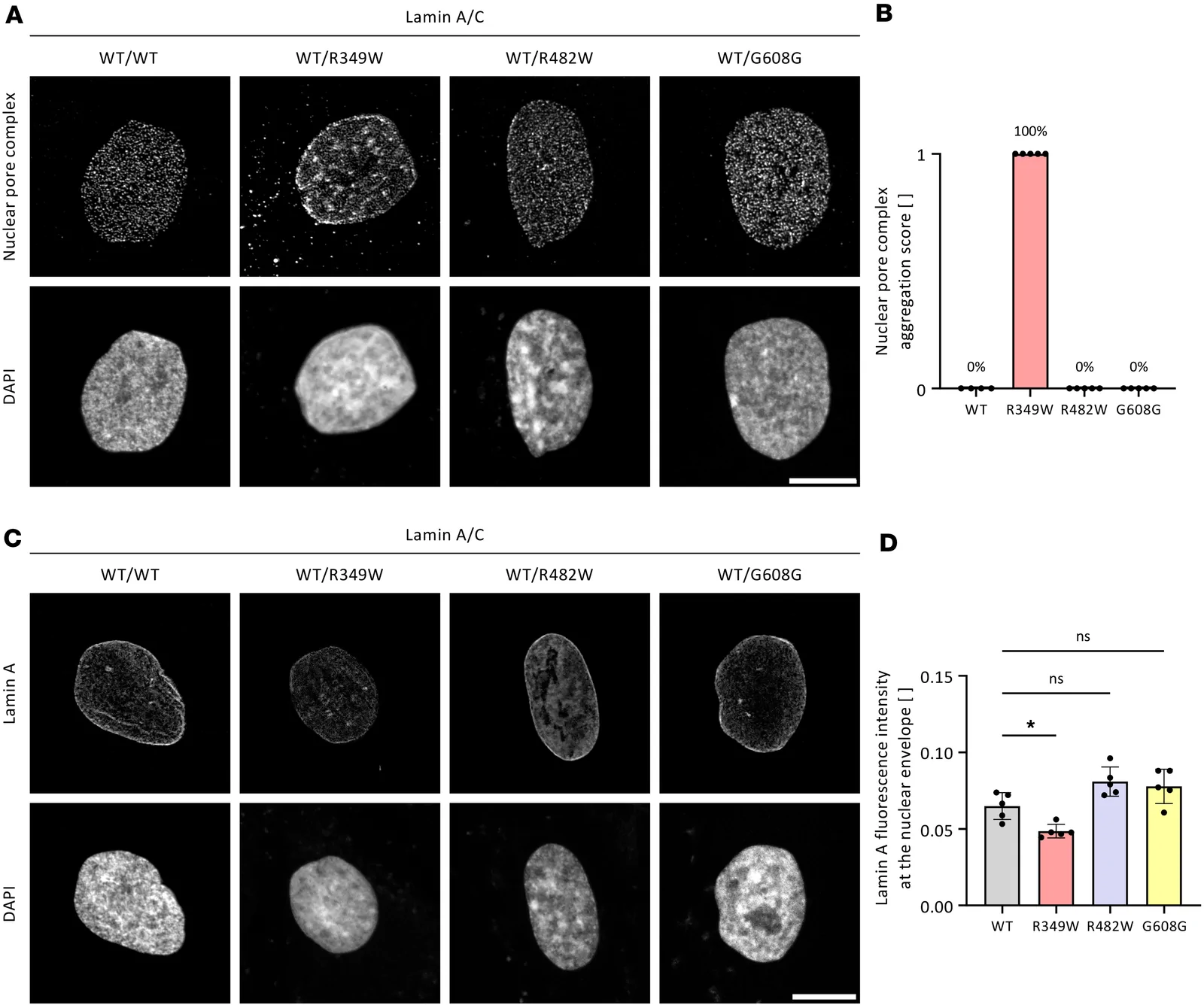
Lamin A/C Arg349Trp localizes to the nuclear envelope and associates with nuclear pore complex aggregation. (**A**) Stimulated emission depletion (STED) micrographs of nuclei from patient-derived dermal fibroblasts harboring WT or mutant lamin A/C as indicated. Nuclear pore complex aggregation was only evident in cells with lamin A/C Arg349Trp. 2 more nuclei per condition are shown in Supplemental Figure 2. Scale bar: 10 μm. (**B**) Quantification of nuclei with nuclear pore complex aggregation as exemplified in **A**. Aggregation score: 0 — no aggregation and 1 — aggregation of nuclear pore complexes. (**C**) STED micrographs as in **A** showing reduced lamin A staining at the nuclear envelope in cells harboring lamin A/C Arg349Trp. Scale bar: 10 μm. (**D**) Quantification of lamin A fluorescence intensity at the nuclear envelope of nuclei exemplified in **C**. Welch’s ANOVA with Dunnett’s T3 multiple comparisons test; mean ± SD. ns, not significant; *P < 0.05.

As lamins A and C appear to be key factors in the process of NPC distribution (27), we next used super-resolution (stimulated emission depletion, STED) microscopy to characterize NPC distribution patterns in primary fibroblasts derived from human skin biopsies. Whereas NPCs were homogenously and evenly scattered across the nuclear envelope in fibroblasts harboring lamin A/C WT, Arg482Trp, or Gly608Gly, cells expressing lamin A/C Arg349Trp consistently exhibited NPC aggregation (Figure 3, A and B, and Supplemental Figure 2). These findings suggest that the Arg349 residue of lamin A/C is critical for proper NPC spacing. We remark that NPC aggregation was described previously for nuclei from primary dermal fibroblasts harboring lamin A/C Gly608Gly but only for later cell passages (28). Of note, lamin A fluorescence intensity at the nuclear envelope was significantly lower in lamin A/C Arg349Trp harboring fibroblasts (Figure 3, C and D).

An increased number of DNA double-strand breaks and DNA damage response activation are commonly observed molecular features in patients with progeroid syndromes due to different *LMNA* mutations (29). Therefore, analysis of 53BP1 and γH2AX, 2 DNA damage response markers, was performed in control and patient dermal fibroblasts harboring lamin A/C Arg349Trp, Arg482Trp, or Gly608Gly (Supplemental Figure 3A). The frequency of cells with 53BP1 or γH2AX foci in lamin A/C Arg349Trp fibroblasts was comparable with that of lamin A/C Gly608Gly fibroblasts, showing a trend toward an increase in DNA damage response when compared with controls. However, it was less pronounced than in fibroblasts from individuals with the lamin A/C Arg482Trp substitution (Supplemental Figure 3, B and C).

### Lamin A/C Arg349Trp impairs the protein’s structural interaction with nucleoporin 155.

**Figure 4:**
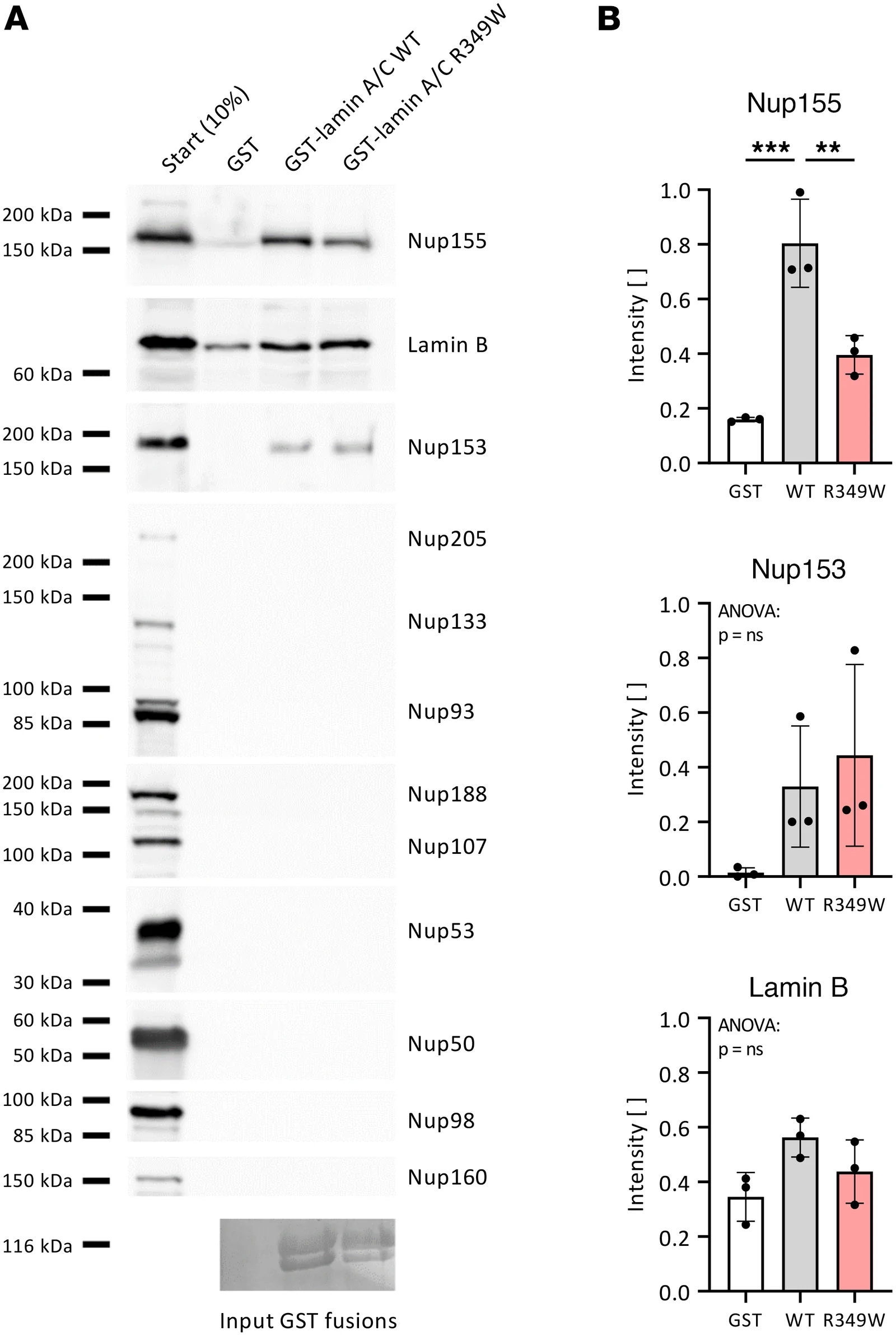
The Arg349Trp substitution in lamin A/C impairs the structural interaction between lamin A/C and Nup155. GST or GST fusion constructs of WT human lamin A/C or the Arg349Trp substitution were incubated with *Xenopus laevis* egg extracts. (**A**) Starting material, corresponding to 10% of the material employed in the pulldowns, as well as bound proteins were analyzed by immunoblotting as indicated. (**B**) Quantification of **A**, showing the average Nup155, lamin B, and Nup153 bead bound signal from 3 independent experiments, normalized to the signal from the starting material. 1-way ANOVAs with Bonferroni-corrected post hoc tests; mean ± SD. ns, not significant; **P < 0.01; ***P < 0.001.

In light of the NPC aggregation observed in lamin A/C Arg349Trp expressing primary dermal fibroblasts (Figure 3A), we next studied potential defects in the structural interaction between lamin A/C Arg349Trp and NPCs. To this end, we probed the interaction between nucleoporins 50, 53, 93, 98, 107, 188, 133, 153, 155, 160, or 205 and lamin A/C Arg349Trp through glutathione S-transferase pulldown of nucleoporins in *Xenopus laevis* egg extracts. Consistent with previous reports (30–32), interactions with Nup155, Nup153, and lamin B could be detected in the lamin A/C WT pulldowns (Figure 4A). From all the nucleoporins tested, there was a uniquely defective interaction between lamin A/C Arg349Trp and Nup155 (Figure 4, A and B). This finding may indicate a critical role for the interaction between Nup155 and lamin A/C in the proper spacing of NPCs.

Of note, Arg349 is located in a coiled-coiled region of the lamin A/C dimer. As the Arg349Trp substitution introduces a hydrophobic residue at this position, the mutation may be expected to interfere with protein homodimerization, as shown by the 3-dimensional illustration of the affected lamin A/C dimer region (Supplemental Figure 4) (33).

### Lamin A/C Arg349Trp associates with alterations of TGF-β signaling.

**Figure 5:**
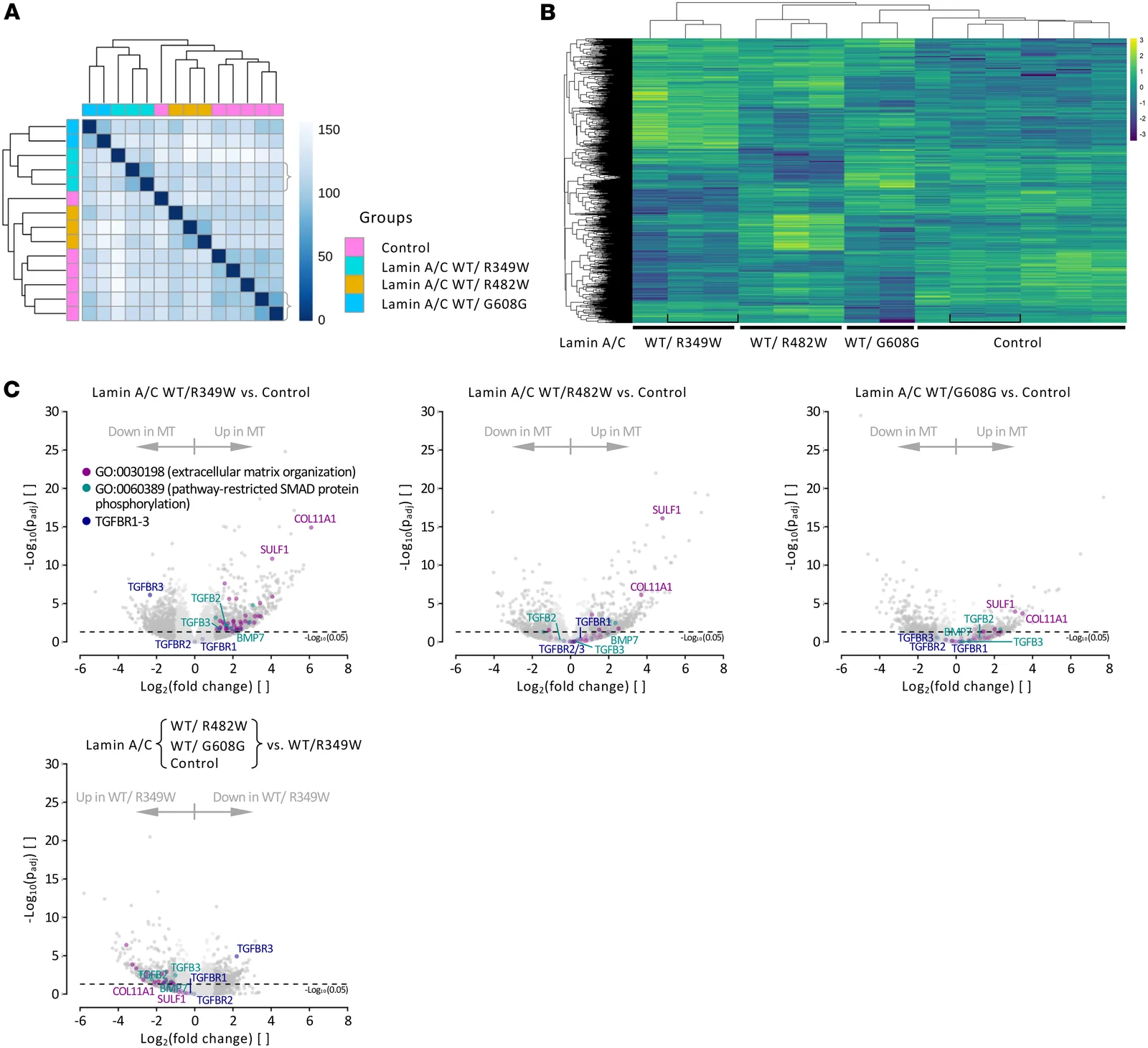
Transcriptomic profiling of patient-derived fibroblasts indicates alterations of TGF-β signaling in cells harboring lamin A/C Arg349Trp. (**A**) Hierarchical clustering analysis of transcriptomic signatures recapitulated the grouping by *LMNA* genotypes. Data derived from biological replicates of the same cell line are indicated by braces. (**B**) Heatmap of significantly differentially expressed genes across all patient-derived fibroblast lines according to the likelihood-ratio test. Hierarchical clustering recapitulated the underlying *LMNA* genotypes. The differential gene expression signature of cells harboring lamin A/C Arg349Trp was markedly different from the signatures of all other genotypes. Data derived from biological replicates of the same cell line are indicated by brackets. (**C**) Volcano plots showing differential gene expression between individual lamin A/C mutant and control cell lines, or between lamin A/C Arg349Trp harboring cells and all other genotypes. Highlighted are selected genes of the color-coded groups identified from the comparison of lamin A/C Arg349Trp harboring and control cell lines. Of note, the pattern of *TGFB2/3* and *BMP7* upregulation together with a reduction in *TGFBR3* expression was only found in this comparison and was recapitulated by comparing all other cell lines with lamin A/C Arg349Trp positive cell lines, with the exception that the change in *BMP7* expression was not significant. MT, mutant.

To probe alterations in signaling pathways associated with lamin A/C Arg349Trp, we obtained bulk RNA-seq data from primary fibroblast cultures derived from human skin biopsies from 2 patients with lamin A/C Arg349Trp, 3 patients with the Arg482Trp substitution, 2 patients with the Gly608Gly mutation, and 5 nonaffected controls. Hierarchical clustering analysis of the transcriptomic signatures recapitulated the grouping by *LMNA* genotypes (Figure 5A). Hierarchical clustering on genes significantly differentially expressed across all patient-derived fibroblast lines revealed that the differential gene expression signature of cells harboring the lamin A/C Arg349Trp variant diverged from those of all other cell lines (Figure 5B). In addition, this hierarchical clustering recapitulated the underlying *LMNA* genotypes, too.

Among the gene ontology (GO) biological processes significantly enriched in genes that were significantly differentially upregulated in lamin A/C Arg349Trp positive versus control cell lines, the highest-ranking terms were *extracellular matrix organization* (GO:0030198) and *extracellular structure organization* (GO:0043062) (Supplemental Figure 5), consistent with the fibrotic processes observed in FSGS. Of note, these biological processes were not found to be ranking high in any other comparison between lamin A/C mutation positive and control cell lines.

In podocytopathies due to *NUP93* mutations, the structural interaction between NUP93 and SMAD4 was shown to be abrogated and bone morphogenetic protein 7 (BMP7)-induced SMAD signaling was demonstrated to be disrupted (13). To assess whether similar alterations in pathways regulated by the TGF-β superfamily occur in lamin A/C Arg349Trp harboring versus control cell lines, we examined the genes from the significantly enriched biological process *pathway-restricted SMAD protein phosphorylation* (GO:0060389) as well as the expression of *TGFBR1*, *TGFBR2*, and *TGFBR3*, which encode the TGF-β receptors 1–3 (Figure 5C). The transcripts corresponding to TGF-β2, TGF-β3, and BMP7 were upregulated in lamin A/C Arg349Trp positive cell lines, together with a reduction in *TGFBR3* expression. This configuration was not found in any other comparison between mutant lamin A/C positive and control cell lines and was recapitulated by comparing all other cell lines with lamin A/C Arg349Trp harboring lines, except for a nonsignificant change in *BMP7* expression.

### Lamin A/C Arg349Trp compromises TGF-β1–dependent SMAD signaling.

**Figure 6:**
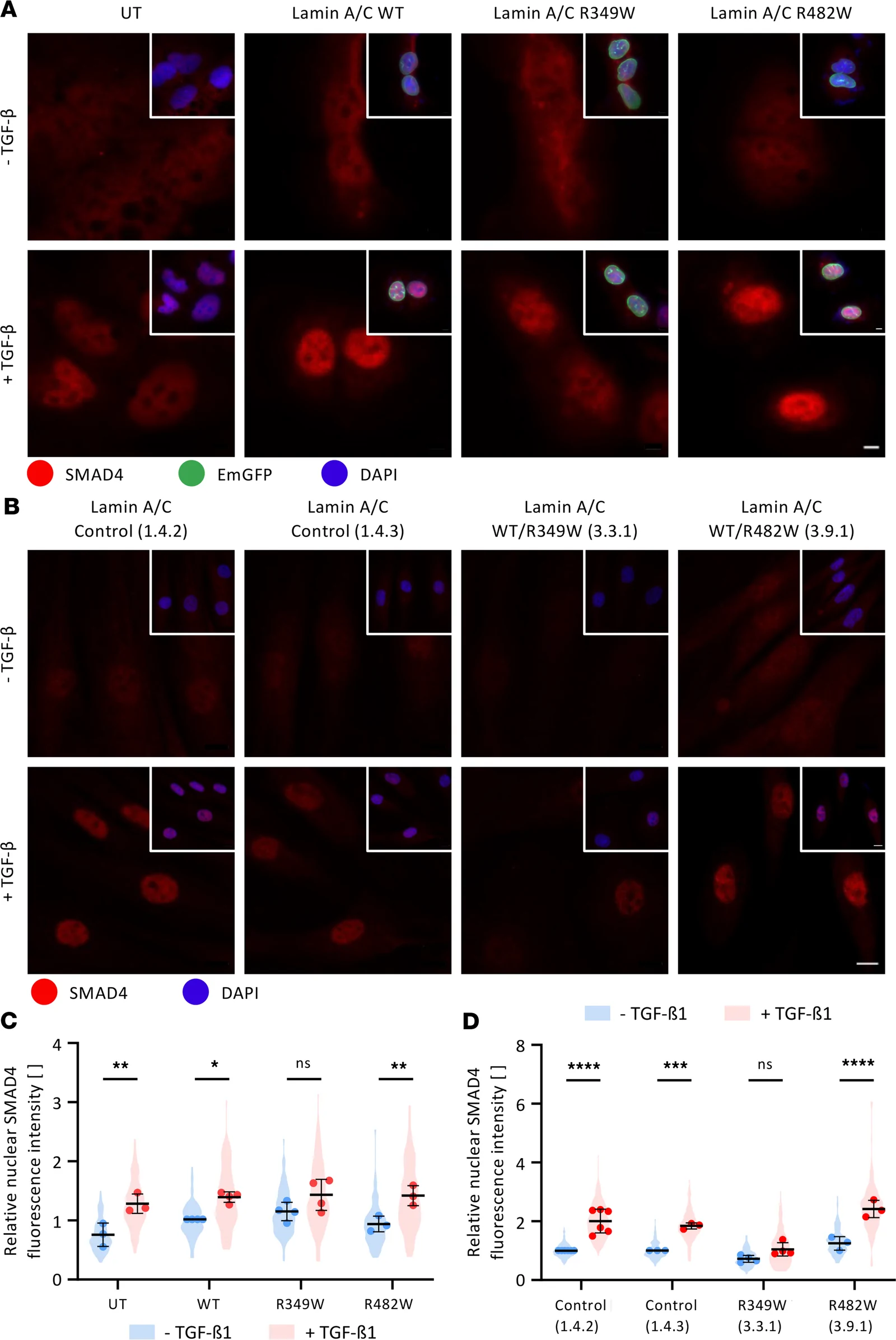
In podocytes and patient-derived fibroblasts, lamin A/C Arg349Trp impedes TGF-β1–induced SMAD4 translocation to the nucleus. Qualitative and quantitative assessments of nuclear SMAD4 accumulation upon TGF-β1 stimulation in immortalized human podocytes transiently transfected with EmGFP-tagged *LMNA* constructs as indicated and in patient-derived dermal fibroblasts. (**A**) Podocytes expressing lamin A/C Arg349Trp showed less pronounced and inconsistent SMAD4 nuclear import upon stimulation with TGF-β1. SMAD4 signal (red) was corrected for nonspecific staining. Nuclei were stained with DAPI (blue). Scale bars: 5 μm. (**B**) Similarly, fibroblasts with heterozygous lamin A/C Arg349Trp showed less pronounced and inconsistent SMAD4 translocation into the nucleus upon stimulation with TGF-β1. SMAD4 signal (red) was corrected for nonspecific staining. Nuclei were stained with DAPI (blue). Scale bars: 20 μm. (**C**) In podocytes expressing lamin A/C Arg349Trp, nuclear SMAD4 fluorescence intensity did not significantly change upon stimulation with TGF-β1. 2-way ANOVA with Bonferroni-corrected post hoc tests; data normalized to mean of unstimulated WT data; mean ± SD. (**D**) In fibroblasts harboring lamin A/C Arg349Trp, nuclear SMAD4 fluorescence intensity did not significantly change upon stimulation with TGF-β1. 2-way ANOVA with Bonferroni-corrected post hoc tests; data normalized to mean of unstimulated control data; mean ± SD. CTRL, control; (∙), (subject identifier); UT, untransfected control; ns, not significant; *P < 0.05; **P < 0.01; ***P < 0.001; ****P < 0.0001.

Next, we aimed to determine whether these enhanced expressions of TGF-β ligands are in line with compensatory ligand upregulation due to disruption of downstream signaling or, rather, indicate pronounced activation of such signaling. To thus probe the integrity of TGF-β1–dependent signaling, we assessed the nuclear accumulation of SMAD4 upon TGF-β1 stimulation in immortalized human podocytes following transient transfection with WT or mutant *LMNA* constructs as well as in patient-derived dermal fibroblasts (Figure 6, A–D). In untransfected podocytes and in those expressing exogenous lamin A/C WT or Arg482Trp, SMAD4 accumulated in the nucleus upon TGF-β1 administration, an effect less pronounced in podocytes expressing lamin A/C Arg349Trp (Figure 6, A and C). These findings were corroborated in primary dermal fibroblasts. Whereas cells harboring lamin A/C WT or Arg482Trp exhibited consistent SMAD4 translocation from the cytosol to the nucleus, this effect was less marked in lamin A/C Arg349Trp fibroblasts (Figure 6, B and D). Thus, in both cell models, the responsiveness of the probed signaling route to TGF-β1 was consistently diminished.

Our data thus indicate alterations of TGF-β ligands and type 3 receptor, which might be compensatory changes due to dysfunctional downstream SMAD-dependent signaling.

## Discussion

Through analysis of unbiased renal outcome data from a large laminopathy patient cohort, we demonstrate here that, unlike typical FPLD as represented by amino acid changes of the hotspot residue Arg482, carriers of the lamin A/C Arg349Trp variant are at high risk of developing nephrotic range proteinuria, progressive eGFR decline, and kidney failure in midlife as part of their multifaceted syndrome. We moreover demonstrate pathogenicity of Arg349Trp by showing that the variant associates with (a) an altered transcriptional profile of affected primary cells, (b) aggregation of their nuclear pore complexes, (c) an abrogation of the structural interaction between lamin A/C and NUP155 in *Xenopus laevis* egg extracts, and (d) an impairment of TGF-β1–dependent SMAD signaling in cultured podocytes and primary fibroblasts. In particular, we show for the first time to our knowledge in a rigorous fashion that lamin A/C Arg349Trp specifically affects the kidney, rendering it the first lamin A/C mutation to cause a genuine kidney phenotype independent of systemic disease manifestations such as diabetic or cardiovascular end-organ damage.

Mechanistically, our data show that TGF-β1 signaling is dysfunctional in lamin A/C Arg349Trp–expressing podocytes, which might be only 1 select instance of a broader defect in cellular signaling through the TGF-β superfamily in podocytopathies due to compromised NPC function. In fact, previous findings in podocytopathies due to *NUP93* mutations support this notion: Braun et al. (13) identified the disruption of BMP7-induced SMAD signaling as a pathogenic mechanism underlying FSGS. Furthermore, via SMAD signaling, podocyte-derived TGF-β1 seems to play a decisive role in podocyte decay and local scarring (34). Like NUP93, NUP155 is a nucleoporin of the inner structural ring of NPCs and crucial for NPC structure and function (35–37). Recently, *NUP155* mutations have been reported in patients with FSGS and mechanistically shown to compromise NPC assembly (38).

Lamins A and C, in turn, have been implicated in TGF-β1–mediated cell cycle control (39). Thus, the intimate functional relationship between nuclear lamina, NPCs, and TGF-β1 signaling may be critical for podocyte survival, and an imbalance of TGF-β1–dependent signaling, as observed in this study, may elicit unique susceptibilities in these cells. In fact, it has been demonstrated that TGF-β1 can induce cell cycle reentry in podocytes; however, their attempts to complete cell division remain unsuccessful, resulting in apoptosis — a phenomenon known as mitotic catastrophe (40). Opposite effects of TGF-β1 on cell cycle regulation and homeostasis have been described for other postmitotic cells. In cardiomyocytes, the antagonization of TGF-β1 contributes to the reinitiation of mitosis (41). In neurons, TGF-β1 promotes cell survival and rescue alongside other members of the TGF-β superfamily (42). These inverse functional roles of TGF-β1 in the cell cycle control of podocytes and in that of cardiomyocytes or neurons highlight the particular vulnerability of podocytes to an imbalance of TGF-β1–dependent signaling.

As a limitation of our study, the model of immortalized podocyte cultures has been controversially discussed (43). Although we provide a comprehensive data set of cellular and clinical information from affected patients, the kidney-specific pathogenic mechanisms of lamin A/C Arg349Trp require further investigation using genetically engineered in vivo models.

As 67% (*n* = 16/24) of the patients with lamin A/C Arg349Trp were reported to develop features of adult-onset progeria (Table 1), the question arises whether the observed renal phenotype in fact constitutes premature aging of the kidney. For podocytes, this hypothesis seems to be all the more relevant as they have been decisively implicated in the development of focal global glomerulosclerosis (FGGS) in healthy aging (44). Beyond podocytes, lamin A/C was also reported to play a critical role in the renal tubular compartment, interfering with tissue repair, cell proliferation, ciliogenesis, and inflammation (45, 46).

From a clinical point of view, our data as well as previously published reports on lamin A/C Arg349Trp associated nephropathy extend the spectrum of laminopathies to also include genuine kidney involvement. As a corollary, we suggest screening patients with pathogenic *LMNA* variants for indicators of kidney disease, notably microalbuminuria as an early marker of podocyte damage. For kidney protection, we suggest timely initiation of treatment with sodium-glucose cotransporter-2 inhibitors and renin-angiotensin-system blockers in microalbuminuric individuals with lamin A/C Arg349Trp, as these substances have proven most beneficial in slowing down CKD progression (47). Additionally, glucagon-like peptide 1 receptor agonists, such as semaglutide, may provide additional kidney protection, given the deranged metabolic profile and developing insulin resistance of affected patients (48). Conversely, genetic testing panels used for diagnosing inherited (proteinuric) kidney diseases should also include *LMNA* as a gene of interest. The insights into the variant-specific clinical implications of genetic *LMNA* alterations provided here, however, are key for accurate variant interpretation and subsequent counseling.

In summary, we have shown that the lamin A/C Arg349Trp variant is linked to kidney disease, which should thus be added to the list of genotype-specific laminopathy manifestations.

## Methods

### Sex as a biological variable.

This study examined clinical data from both male and female patients, and we show that the reported findings are independent of sex.

### Genetic analysis.

In the lamin A/C Arg349Trp patient identified in Germany, whole exome sequencing (WES) was performed in the patient and both of her parents. Next generation sequencing (NGS) was performed according to the manufacturer’s instructions. In brief, genomic DNA was extracted from blood lymphocytes by a standard protocol (QIAGEN). Library preparation was conducted according to the manufacturer’s protocol (Twist Bioscience). Precapture amplified samples were pooled and hybridized against the Twist Exome RefSeq Kit (Twist Bioscience). NGS was performed on an Illumina system. Variant interpretations of the WES data followed strict internal guidelines with a focus on genes associated with the disease of interest. Sequence variants of interest were verified by Sanger sequencing if NGS results failed internal validation guidelines.

In the patients identified in France, the *LMNA* p.(Arg349Trp) variant was detected by direct Sanger sequencing of exons and flanking intronic regions of *LMNA*. Genomic DNA was extracted from peripheral blood leucocytes. Exons and flanking intronic sequences of *LMNA* were amplified by PCR, sequenced with the BigDye Terminator sequencing kit (Applied Biosystems) and run on an automated sequencer. Sequences were analyzed with SeqScape (Applied Biosystems).

### Expression constructs.

WT *LMNA* with N-terminal EmGFP or mCherry tags in pcDNA6.2 vectors were kind gifts of Monica Carmosino from the University of Basilicata in Potenza, Italy (49, 50). The point mutations of interest (c.1045C>T, c.1444C>T, and c.1824C>T in the *LMNA* gene, NM_170707.4) were introduced into these constructs using the Q5 Site-Directed Mutagenesis Kit (E0554S, New England Biolabs, Ipswich, MA, USA). Correct mutagenesis was verified by Sanger sequencing.

For glutathione S-transferase (GST) pulldown assays, human WT lamin A/C or the Arg349Trp mutant were cloned into a modified pET28a vector with an N-terminal GST-tag, followed by a TEV cleavage site.

### Cell culture.

Immortalized human podocytes were kindly provided by Daniela A. Braun from the University Hospital Muenster in Muenster, Germany. Podocytes were cultured in RPMI-1640 Medium (R8758, Sigma-Aldrich, St. Louis, MO, USA) supplemented with 10% v/v FBS Superior (S0615, Biochrom, Berlin, Germany), 1% v/v penicillin/ streptomycin (15140122, Gibco, Thermo Fisher Scientific, Waltham, MA, USA), and 1% v/v insulin/ transferrin/ selenium (41400045, Gibco, Thermo Fisher Scientific, Waltham, MA, USA) in a 5% CO_2_ atmosphere at 33°C (permissive conditions).

Human embryonic kidney 293T (HEK293T) cells (DSMZ-German Collection of Microorganisms and Cell Cultures, Braunschweig, Germany) and primary human dermal fibroblasts were cultured in DMEM (Gibco, Thermo Fisher Scientific, Waltham, MA, USA) with 10% v/v FBS Superior (S0615, Biochrom, Berlin, Germany) and 1% v/v penicillin/ streptomycin (15140122, Gibco, Thermo Fisher Scientific, Waltham, MA, USA) in a 5% CO_2_ atmosphere at 37°C.

### Immunofluorescence microscopy.

For immunostaining, we used commercially available fixative, permeabilization solution, and blocking buffer (R37602, Invitrogen, Thermo Fisher Scientific, Waltham, MA, USA). Cells were fixed in 4% formaldehyde in PBS for 15 minutes, washed once with PBS, permeabilized with 0.5% Triton X-100 for 15 minutes, and washed once with PBS. Nonspecific binding sites were blocked with 3% BSA in DPBS for 1 hour. Cells were then incubated in primary antibody diluted in PBS for 3 hours at room temperature. Following one wash with PBS, cells were incubated in secondary antibody in PBS for 1.5 hours at room temperature. After another PBS wash, nuclei were stained with DAPI (R37606, Invitrogen, Thermo Fisher Scientific, Waltham, MA, USA) for 10 minutes before the cells were mounted (50001, ibidi, Graefelfing, Germany). Slides were imaged on an AxioObserver.Z1 microscope with an ApoTome Imaging System (Carl Zeiss, Oberkochen, Germany).

The following antibodies were used: mouse anti-Smad4 (sc-7966, Santa Cruz Biotechnology, Dallas, TX, USA) at 1:50 and Alexa Fluor 568 goat anti-mouse (A11004, Invitrogen, Thermo Fisher Scientific, Waltham, MA, USA) at 1:500.

### Overexpression studies.

Immortalized human podocytes or HEK293T cells were seeded on 8-well μ-slides (80826, ibidi, Graefelfing, Germany) at 60–80% confluence. After 24 hours, when they had reached 90-100% confluence, cells were transiently transfected using Lipofectamine 2000 (11668-019, Invitrogen, Thermo Fisher Scientific, Waltham, MA, USA). Cells were fixed and stained, or experiments were conducted 24 hours after transfection.

For the assessment of nuclear morphologies, cells were transfected with 500 ng plasmid — i.e., 500 ng for single transfections and 250 ng of each construct for double transfections, respectively. Following fixation and immunostaining, abnormalities in nuclear shape, defined as bleb formation or multilobulation, were counted by 2 independent observers blinded to the experimental conditions.

Alterations of TGF-β1–induced Smad4 transition into the nucleus were examined in immortalized human podocytes after transfection with 500 ng plasmid (WT versus mutant *LMNA* containing constructs) and in patient-derived dermal fibroblasts. Dermal fibroblasts were starved in DMEM for 3 hours before TGF-β1 treatment. Cells were stimulated with TGF-β1 (PHG9204, Invitrogen, Thermo Fisher Scientific, Waltham, MA, USA) at 10 ng/mL for 30 minutes before they were fixed and processed for immunostaining.

For all experiments, data from 3 biological replicates were acquired.

### Quantification of nuclear SMAD4.

Images were first corrected for background fluorescence using ZEN light software (Carl Zeiss, Oberkochen, Germany). For each nucleus, Smad4 enrichment was then determined by measuring the mean fluorescence intensity within a predefined circular area entirely contained in the nucleus using ImageJ software (NIH) (51). This area was kept constant throughout all measurements.

### STED microscopy of patient-derived dermal fibroblasts.

For STED microscopy, primary fibroblasts were grown in chambered 8-well coverslips (80827, ibidi, Graefelfing, Germany) in DMEM (10564011, Gibco, Thermo Fisher Scientific, Waltham, MA, USA) supplemented with 10% v/v heat-inactivated FBS (17593595, Gibco, Thermo Fisher Scientific, Waltham, MA, USA) and 1% v/v penicillin/streptomycin (15140122, Gibco, Thermo Fisher Scientific, Waltham, MA, USA). Cells were incubated overnight at 37°C and 5% CO_2_ before they were washed with PBS, fixed with 2% formaldehyde in PBS, quenched with 10 mM NH_4_Cl in PBS, and permeabilized using 0.1% Triton X-100 in PBS. Unspecific sites were blocked by incubation in blocking buffer (2% BSA in PBS) for 1 hour, followed by incubation with primary antibodies diluted in blocking buffer for 1 hour at room temperature. Cells were then washed 3 times with PBS, followed by incubation with fluorescently tagged secondary antibodies diluted in blocking buffer for 1 hour at room temperature. Finally, cells were washed with PBS followed by double distilled water and mounted using Mowiol mounting medium (81381, Sigma-Aldrich, Burlington, MA, USA) supplemented with 1 μg/mL DAPI (6843, Carl Roth, Karlsruhe, Germany). Cells were imaged using an inverted Leica SP8 Tau-STED3X (Leica Biosystems Nussloch, Nussloch, Germany) with a Leica HC PL APO 93x/NA 1.30 glycerol-based STED objective and proper filter settings. Images were visualized using imaris and ImageJ software.

The following primary antibodies were used: mouse anti-nuclear pore complex proteins antibody (clone MAb414, MMS-120R, Covance, NJ, USA) and rabbit anti-lamin A antibody (ab26300, abcam, Cambridge, United Kingdom).

The following secondary antibodies were used: Alexa Fluor 488 goat anti-rabbit (A-11008, Invitrogen, Thermo Fisher Scientific, Waltham, MA, USA), Alexa Fluor 647 goat anti-mouse (A-21235, Invitrogen, Thermo Fisher Scientific, Waltham, MA, USA), and abberior STAR RED goat anti-mouse (STRED-1001-500UG, abberior, Göttingen, Germany) antibodies.

### RNA-seq.

RNA was extracted from pelleted cells using the RNeasy Plus Mini Kit (74134, Qiagen, Hilden, Germany), essentially according to the manufacturer’s instructions. RNA integrity was determined with Agilent’s 4200 TapeStation (Agilent Technologies, Santa Clara, CA, USA). RNA-seq libraries were prepared with 200 ng total RNA input and the TruSeq Stranded mRNA Library Prep assay (Illumina, San Diego, CA, USA) based on the recommended protocol. After poly-A selection (using poly-T oligo-attached magnetic beads), mRNA was purified and fragmented using divalent cations under elevated temperatures. The RNA fragments underwent reverse transcription using random primers. Following second-strand cDNA synthesis with DNA Polymerase I and RNase H, end repair, and A-tailing, indexing adapters were ligated. The products were then purified and amplified (15 PCR cycles) to create the final cDNA libraries which were validated and quantified (4200 TapeStation, Agilent Technologies, Santa Clara, CA, USA). Equimolar amounts of the libraries were pooled and quantified using the Peqlab KAPA Library Quantification Kit and the 7900HT Sequence Detection System (Applied Biosystems, Foster City, CA, USA). Sequencing was done with an Illumina NovaSeq 6000 sequencer (Illumina, San Diego, CA, USA) with a paired-end 101 bp read length aiming at 50 million reads per sample. Demultiplexing and FASTQ file generation were performed using Illumina’s bcl2fastq2 software (v2.20.0).

### RNA-seq data analysis.

RNA-seq data analysis was performed with the Cologne Center for Genomics’ inhouse pipeline for RNA-seq analysis based on the Nextflow domain-specific language (v20.01.0, build 5264) (52). In brief, sequence reads were adapter-trimmed with Trimmomatic (v0.38) (53), removing sequences of length < 18 nt after trimming, before they were mapped against the hg38 human reference genome and the gene assembly v101 using STAR aligner (v2.6.1a) (54) with the default settings. Subread (v1.6.4) (55) was used to generate the count matrix. Differentially expressed genes (DEGs) were called using DESeq2 (v1.20.0) (56) with a *P*_adj_ cutoff of 0.05. Overrepresentation of GO biological processes among up- or down-regulated DEGs (with *P*_adj_ < 0.05 and log_2_ fold change > 2 for either up or down-regulated DEGs) was determined using clusterprofiler (v3.17.0) (57). Volcano plots were generated with ggplot2 (v3.5.2) (58). For likelihood-ratio testing, raw counts were analyzed using DESeq2 (v1.48.1) (56) with a *P*_adj_ cutoff of 0.05. For heatmap generation, DESeq2 normalized data for genes with *P*_adj_ < 0.05 according to the likelihood-ratio test were log_2_ transformed, *z* scored across samples and visualized with pheatmap (v1.0.13) (https://CRAN.R-project.org/package=pheatmap). Other heatmaps were generated with the heatmap.2 subfunction of gplots (v3.1.0) or with pheatmap (v1.0.12), respectively (https://github.com/talgalili/gplots; commit ID 3da455d, and https://github.com/raivokolde/pheatmap; commit ID b333453, respectively).

### Nucleoporin pull-down assay.

For pull-down assays, GST bait proteins (GST only, GST lamin A/C WT, and GST lamin A/C Arg349Trp) were expressed in BL21de3 *E*. *coli* by autoinduction in LB medium at 18°C and purified using Ni-NTA beads (Qiagen). At 0.5 μM, these GST bait proteins were incubated with 400 μL of *Xenopus laevis* egg extracts for 1 hour at 4°C, supplemented with 60 μL of 50% GSH-Sepharose 4B (GE Healthcare) slurry, and incubated for another 2 hours. The Sepharose was washed 5 times with PBS, and bead-bound proteins were eluted in 30 μL total volume by TEV protease cleavage (0.5 mg/mL) for 1 hour at 25°C. The TEV protease cleaves the GST fusions between the GST moiety and the bait proteins. The input and elutions were analyzed by Western blotting using previously characterized antibodies specific for *Xenopus laevis* proteins at 1:1,000 dilutions — Nup50 (59); Nup93 (37); Nup53, Nup98, Nup107, Nup188, and Nup205 (60); Nup133, Nup153, and lamin B (61); and Nup155 and Nup160 (62). For quantification, enhanced chemiluminescence signals were determined using FiJi. Background signals were subtracted, and the signal ratio of eluate/ input was calculated.

### Statistics.

Statistical analyses were performed as indicated using GraphPad Prism software (GraphPad Software, La Jolla, CA, USA; the latest version used was 11.0.2, build 92). Statistical significance was assumed at *P* < 0.05.

### Study approval.

Written informed consent was obtained from all study participants prior to participation. Where applicable, patients gave written informed consent for the use of their photographs, and the record of informed consent has been retained. The use of patient data and materials was approved by the respective IRBs at the University of Leipzig Medical School (IRB00001750, approval nos. 402/16-ek and 135/13-ek) in Leipzig, Germany; the Saint-Antoine Hospital and the Saint-Antoine Research Center (CCP Ile-de-France 5 and IRB DC-2008-529) in Paris, France; and the Karolinska Institute (Stockholm Department of Medicine 2 IRB, approval no. Dnr 2024-06166-02) in Stockholm, Sweden. For clinical data acquired in Pisa, Italy, ethical approval was granted by the IRB at the University Hospital of Pisa (IRB “Comitato Etico Regionale per la Sperimentazione Clinica della Toscana - Area Vasta Nord Ovest (CEAVNO)”, approval no. 12258). Primary dermal fibroblast cultures derived from patients with HGPS (HGADFN003, HGADFN164, and HGADFN188) were obtained from the Progeria Research Foundation cell and tissue bank.

Apart from single-patient data obtained directly from treating physicians, patient data were also extracted from the ECLip Registry (22). Written informed consent has been obtained for all patients participating in the ECLip Registry unless local ethical approval covered anonymous patient data entry for deceased patients or those lost to follow-up. As the ECLip Registry server is hosted at Ulm University, Ulm University ethic committee is the leading IRB for the registry (approval no. 408/17).

We remark that, for the patient cohorts curated for this study, the obtainable patient datasets varied with respect to the reported clinical data, yielding different cohort sizes for the readouts of interest.

### Data availability.

The data underlying the findings of this study are available in the Supporting Data Values file or from the corresponding author upon request. RNA-seq data can be found in the Gene Expression Omnibus (GEO; https://www.ncbi.nlm.nih.gov/geo/) data repository under the accession no. GSE335536.

## Author contributions

JH and SS conceived the study. CA, SS, M Hamed, GR, and CF performed experiments. CW contributed to data analysis. MK contributed histopathological images and electron micrographs. SS, CA, KM, ST, BA, DAV, GC, ÉC, AG, M Heni, TS, IŠ, ES, MCV, EV, MW, JVS, C Vatier, JR, YR, C Vigouroux, and JH contributed clinical data, which were curated by CA. SS and RS visualized 3D protein structure models. TG performed bioinformatics analyses. CB performed genetic testing. ME and C Vigouroux contributed primary dermal fibroblast lines. ME and WA contributed to experimental designs. ME, WA, and C Vigouroux contributed to study concept. JH supervised the study. SS, CA, and JH wrote the manuscript, which was reviewed and edited by other authors. The order of the 2 co–first authors reflects that SS did more conceptual work.

## Conflict of interest

The authors have declared that no conflict of interest exists.

## Funding support

- German Research Foundation (Deutsche Forschungsgemeinschaft, DFG), project ID 502928386 (SS).
- DFG project ID 508310822 (M Hamed).
- DFG project ID 539950728 and Else Kröner-Fresenius Foundation (2019_A96) (RS).
- DFG project ID 445703531 (WA).
- DFG project IDs 451693578, 471294925, 496611648, and 539950728 (JH).

## Supplementary Material

Supplemental data

ICMJE disclosure forms

Unedited blot and gel images

Supporting data values

## Figures and Tables

**Table 1:**
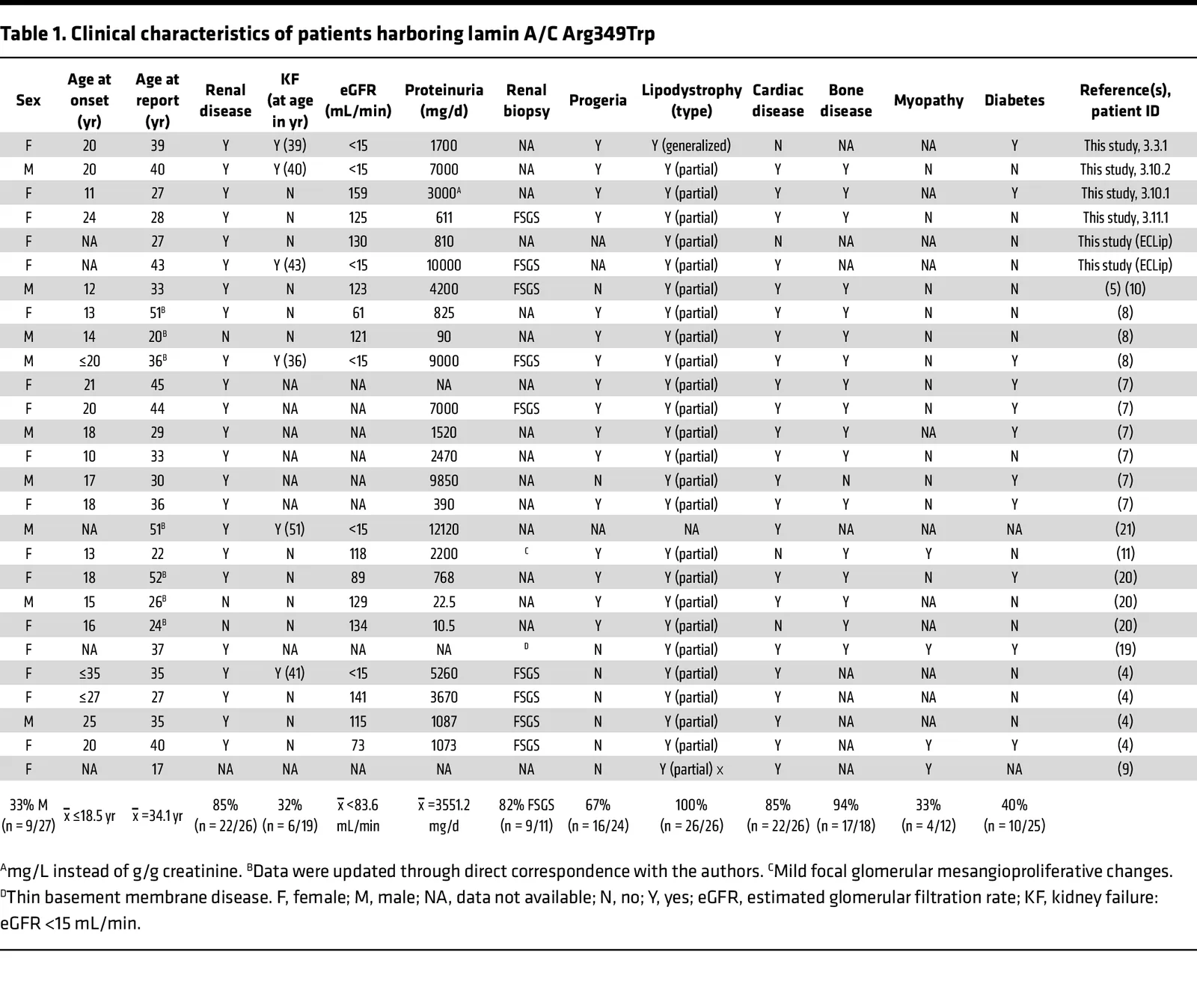
Clinical characteristics of patients harboring lamin A/C Arg349Trp

## Version 1 07/16/2026

In-Press Preview

## Version 2 09/22/2026

Electronic publication
