# Supplementary material for: The risk of nephrotic range proteinuria and kidney failure in primary laminopathies is genotype specific
Source: JCI Insight. 2026 Jul 16;11(18):e192062. doi: 10.1172/jci.insight.192062 (PMC13596725; doi:10.1172/jci.insight.192062)
Supplement: Supplementary file 1 [file jciinsight-11-192062-s062.pdf]

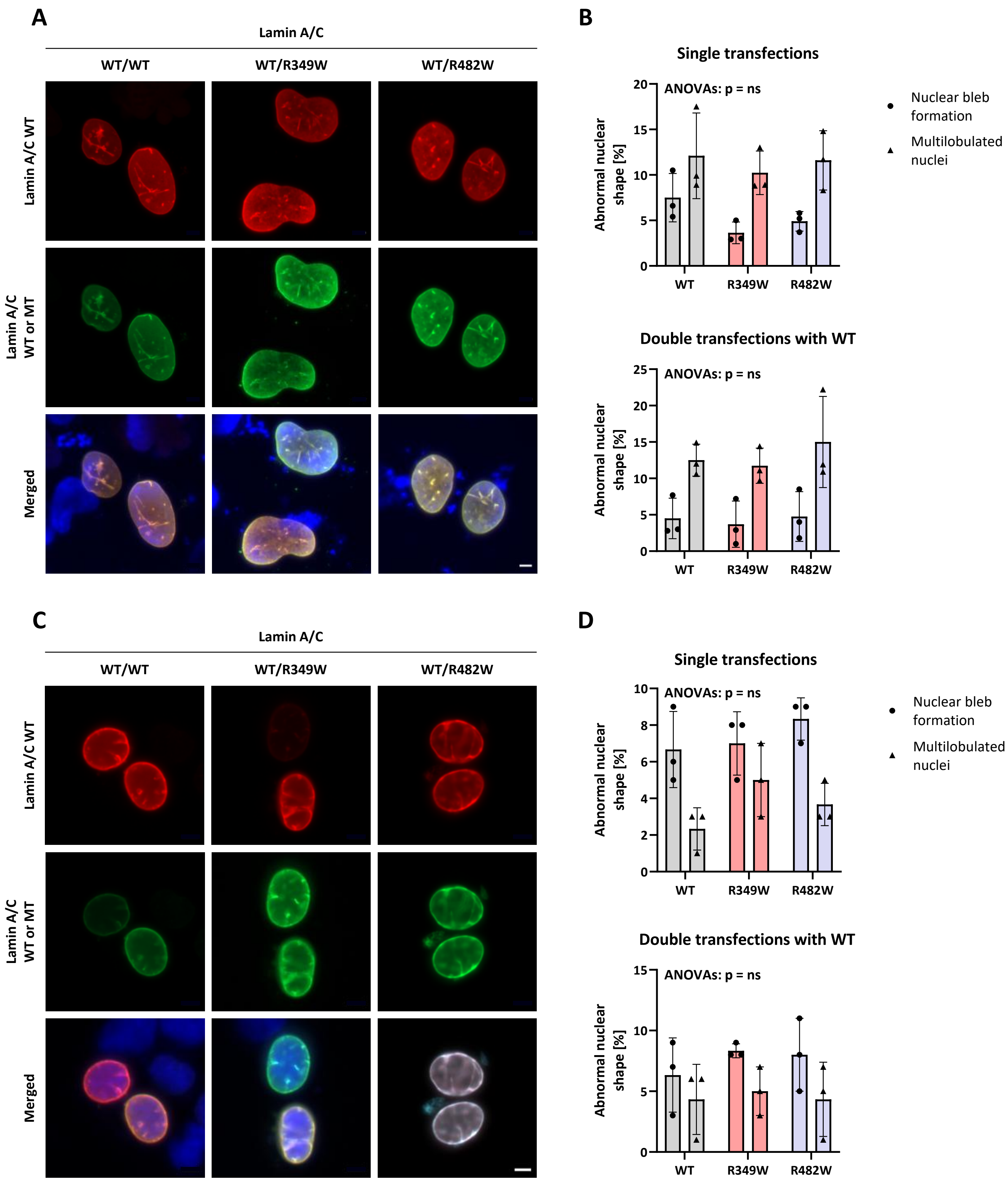

Supplemental Figure 1. Lamin A/C Arg349Trp localizes to the nuclear envelope and does not alter nuclear morphology in immortalized human podocytes and HEK293T cells. Immortalized human podocytes or HEK293T cells were transiently co-transfected with WT mCherry-tagged *LMNA* and WT or mutant EmGFP-tagged *LMNA* constructs as indicated. (**A**) In podocytes, WT and mutant lamin A/C colocalized at the nuclear envelope. Nuclei were stained with DAPI (blue). Scale bar: 5 µm. (**B**) In podocytes, the amount of abnormally shaped nuclei did not significantly differ between *LMNA* genotypes. Data from 1 of 2 observers; 1-way ANOVAs; number of nuclei analyzed, n = 99-121; mean ± SD. (**C**) In HEK293T cells, WT and mutant lamin A/C colocalized at the nuclear envelope. Nuclei were stained with DAPI (blue). Scale bar: 5 µm. (**D**) In HEK293T cells, the amount of abnormally shaped nuclei did not significantly differ between *LMNA* genotypes. Data from 1 of 2 observers; 1-way ANOVAs; number of nuclei analyzed, n = 100-104; mean ± SD. MT, mutant; ns, not significant.

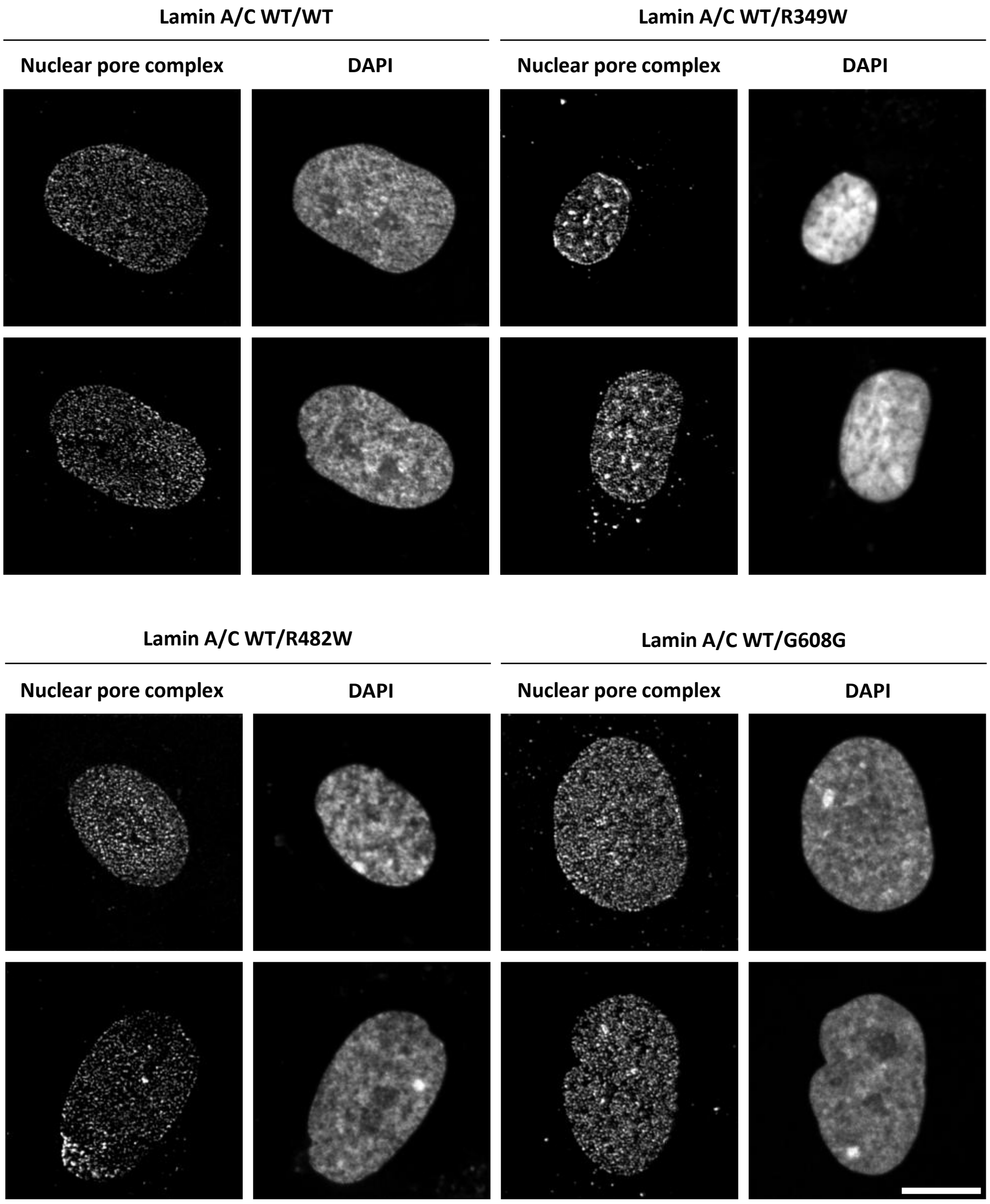

Supplemental Figure 2. Lamin A/C Arg349Trp associates with nuclear pore complex aggregation. Stimulated emission depletion (STED) micrographs of nuclei from patient-derived dermal fibroblasts harboring WT or mutant lamin A/C as indicated. Nuclear pore complex aggregation was only evident in cells with lamin A/C Arg349Trp. In addition to the nuclei shown in **Figure 3A**, 2 more nuclei per condition are presented here. Scale bar: 10 µm.

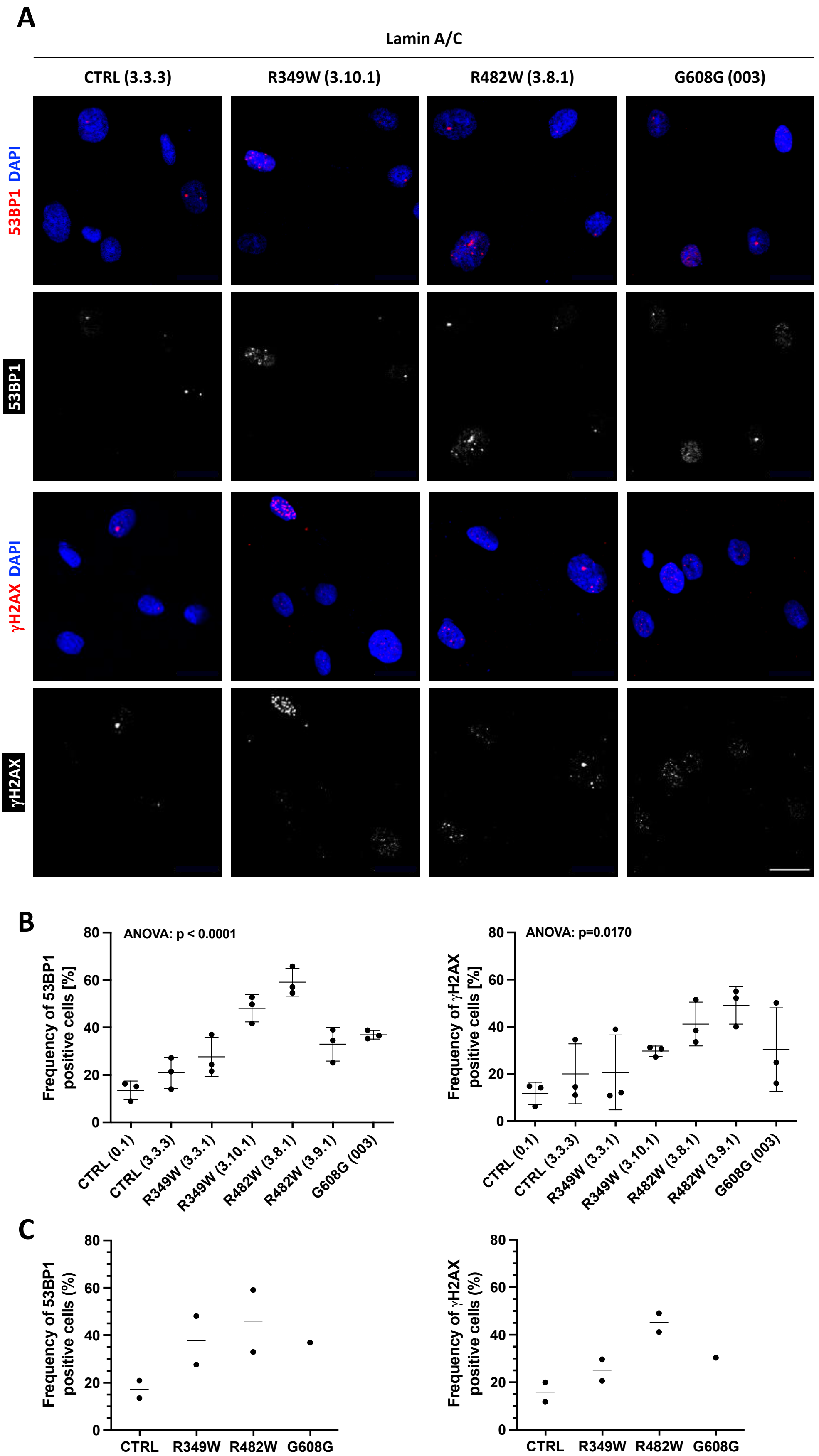

Supplemental Figure 3. (**A**) Immunofluorescence staining of DNA damage response markers 53BP1 and γH2AX (red) in nuclei (blue) from patient-derived dermal fibroblasts harboring WT or mutant lamin A/C as indicated. Scale bar: 25 µm. (**B**) Patient-derived fibroblasts with heterozygous lamin A/C Arg349Trp showed similar DNA damage response activation as control and lamin A/C Gly608Gly fibroblasts. 1-way ANOVAs; number of nuclei analyzed, n = 107-425; mean ± SD. (**C**) Lamin A/C Arg349Trp fibroblasts showed a trend towards an increase in DNA damage response activation compared to control fibroblasts. Triplicates were analyzed for each patient sample (see **B**), and the averages of these triplicates were plotted in **C**. CTRL, control; (·), (subject identifier).

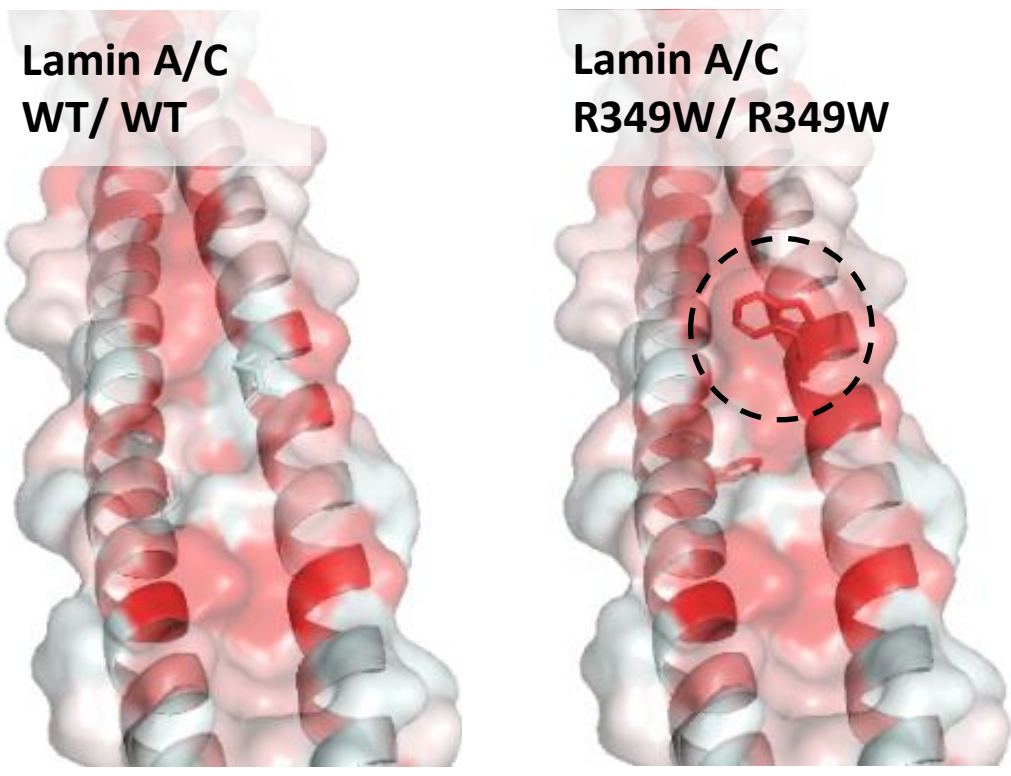

Supplemental Figure 4. Part of lamin A/C dimer structure (PDB ID 1X8Y, Strelkov et al. *J Mol Biol* 2004) with cartoon and surface colored according to hydrophobicity (red), PyMol script Color h.

A

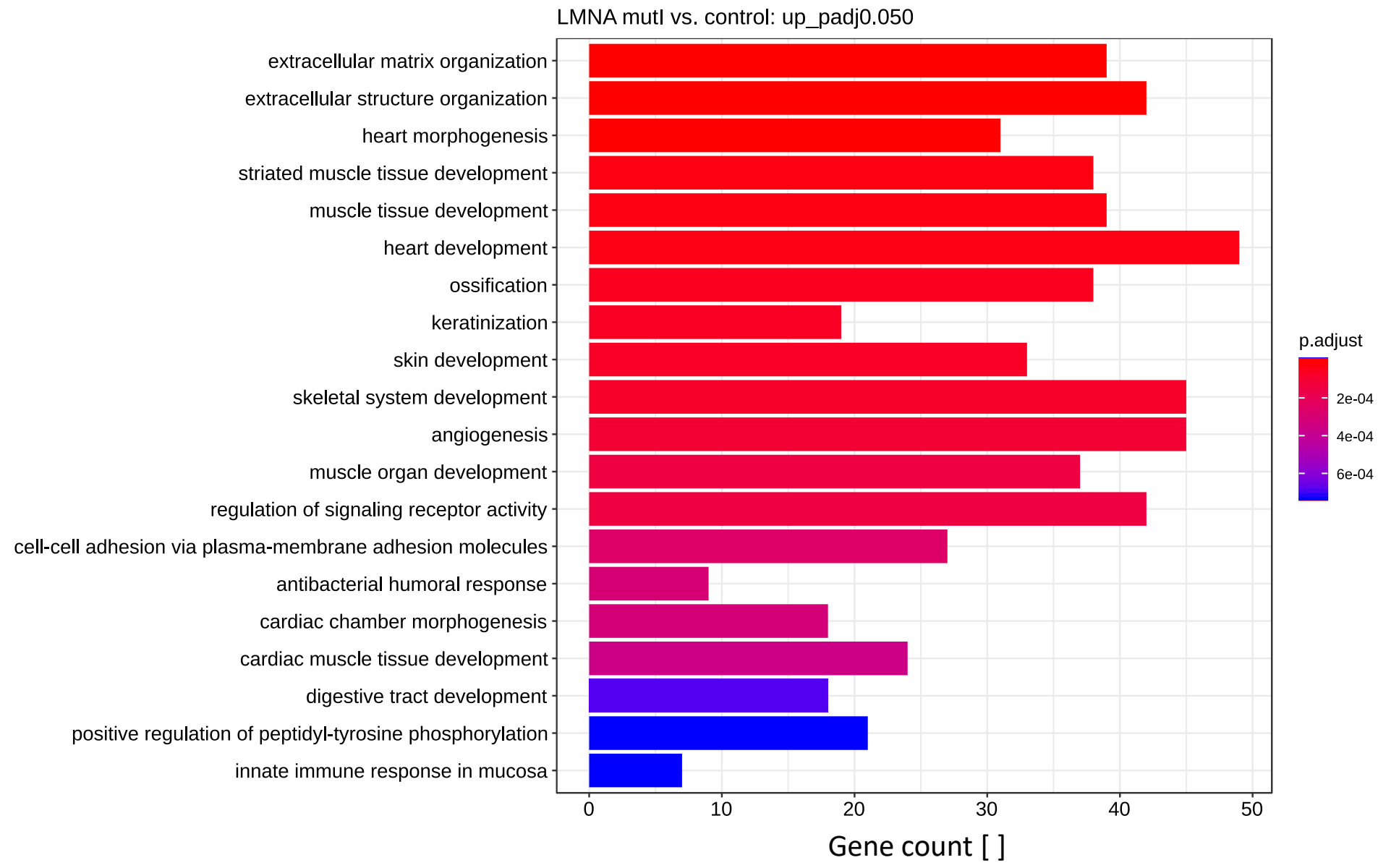

B

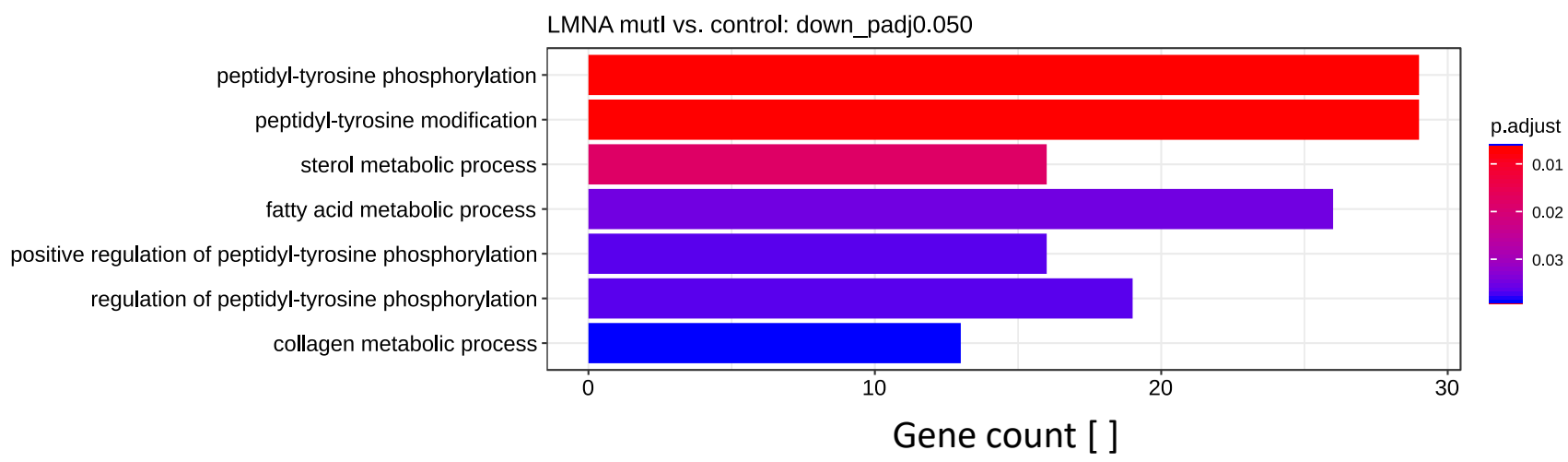

Supplemental Figure 5. Enrichment analysis of gene ontology biological processes in the set of significantly differentially expressed genes between lamin A/C Arg349Trp harboring patient-derived fibroblasts and control lines ($p_{adj} < 0.05$). (**A**) The first 20 of the 297 significantly enriched biological processes in genes significantly differentially upregulated in lamin A/C Arg349Trp positive cells. (**B**) All 7 biological processes significantly enriched in genes significantly differentially downregulated in lamin A/C Arg349Trp harboring cells.
