# Supplementary material for: The risk of nephrotic range proteinuria and kidney failure in primary laminopathies is genotype specific
Source: JCI Insight. 2026 Jul 16;11(18):e192062. doi: 10.1172/jci.insight.192062 (PMC13596725; doi:10.1172/jci.insight.192062)
Supplement: Supplementary file 3 [file jciinsight-11-192062-s064.pdf]

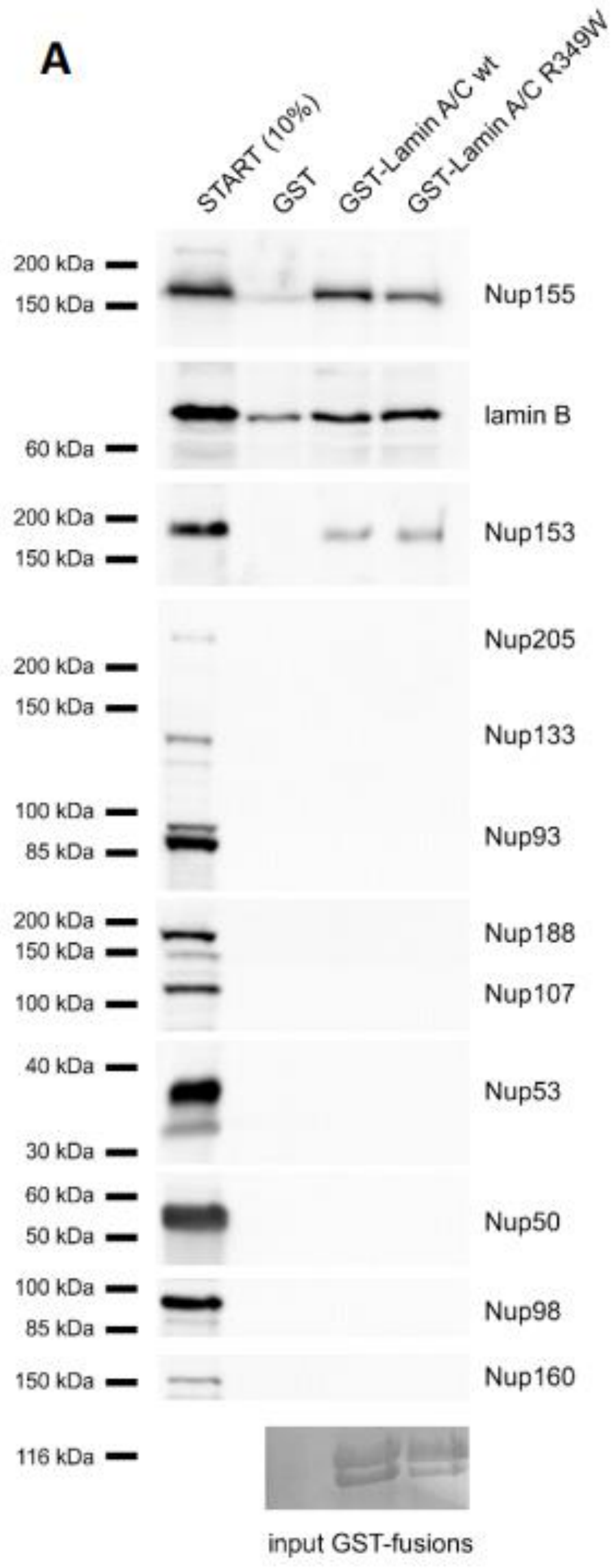
A
START (10%)
GST
GST-Lamin A/C wt
GST-Lamin A/C R349W
200 kDa
150 kDa
Nup155
lamin B
60 kDa
200 kDa
150 kDa
Nup153
Nup205
200 kDa
150 kDa
Nup133
100 kDa
85 kDa
Nup93
200 kDa
150 kDa
Nup188
100 kDa
Nup107
40 kDa
Nup53
30 kDa
60 kDa
50 kDa
Nup50
100 kDa
85 kDa
Nup98
150 kDa
Nup160
116 kDa
input GST-fusions

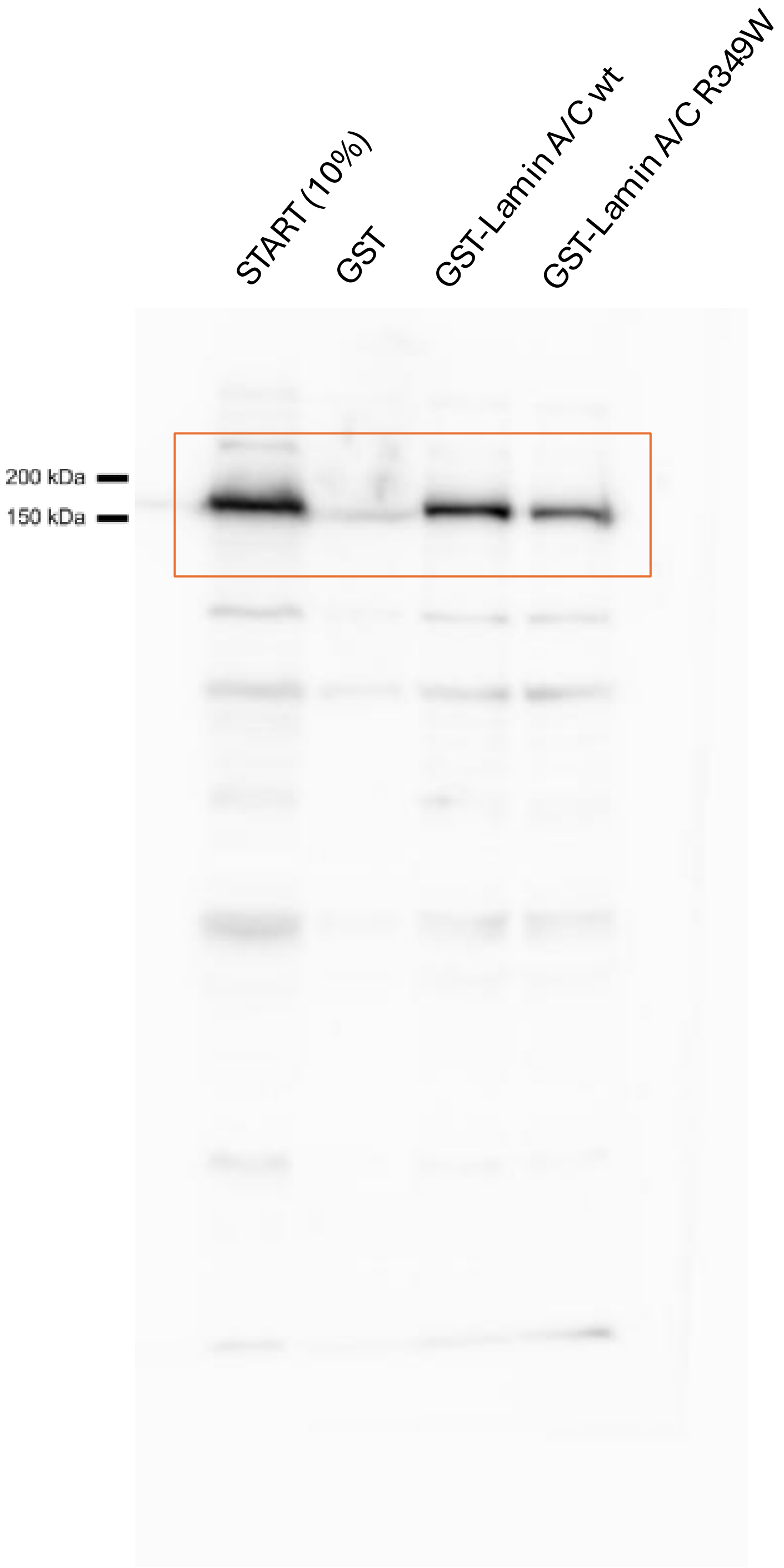

Nup155 band used for figure 4A

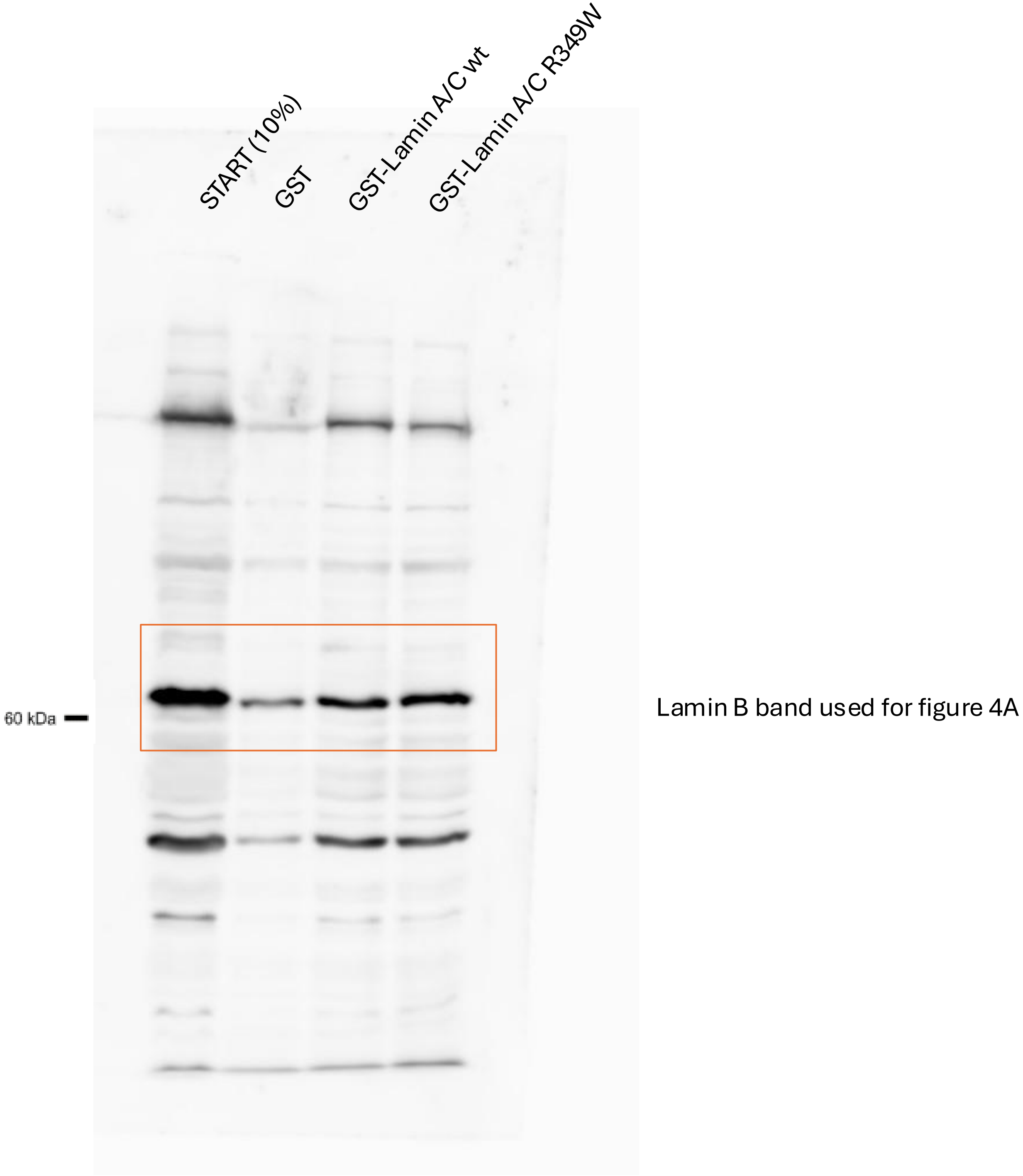
START (10%)
GST
GST-Lamin A/C wt
GST-Lamin A/C R349W
60 kDa
Lamin B band used for figure 4A

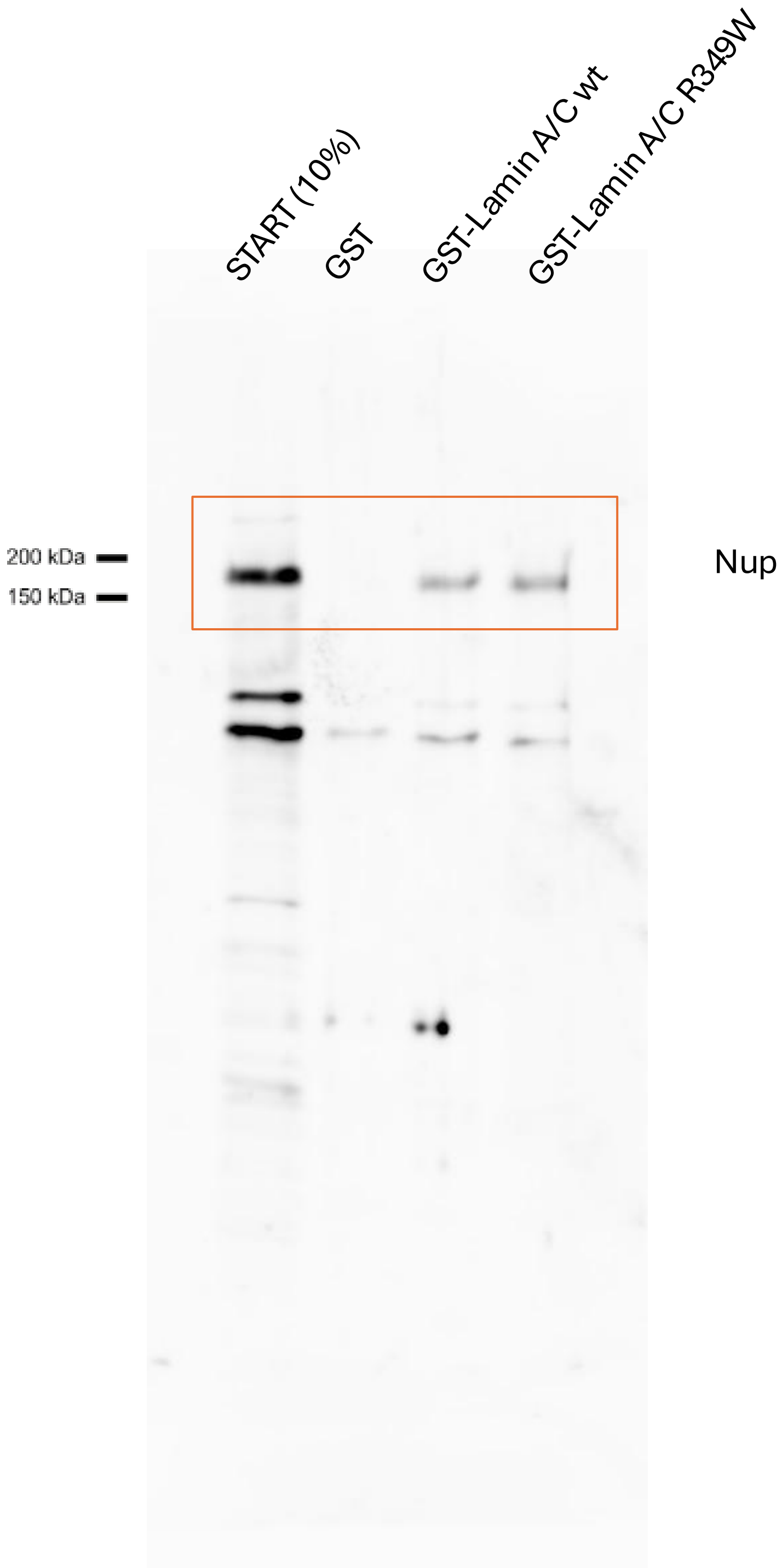

Nup153 band used for figure 4A

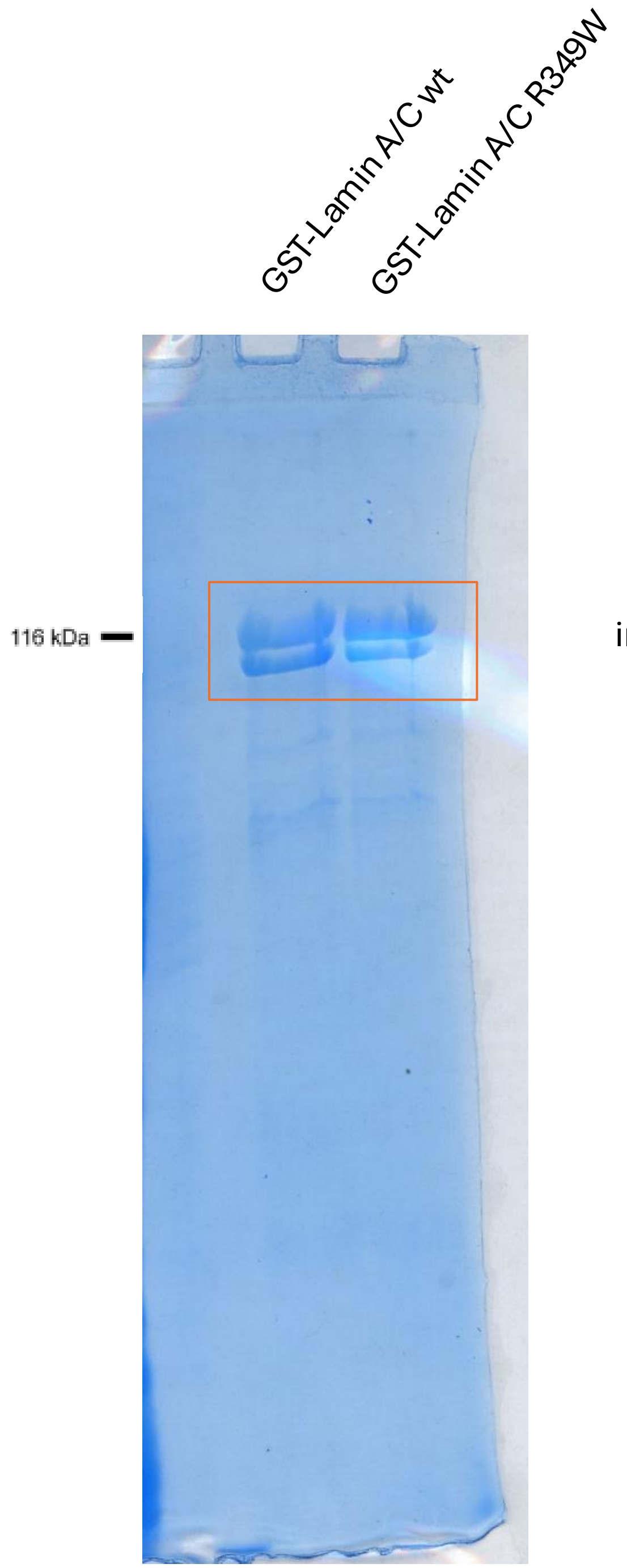

input GST-fusions used for figure 4A

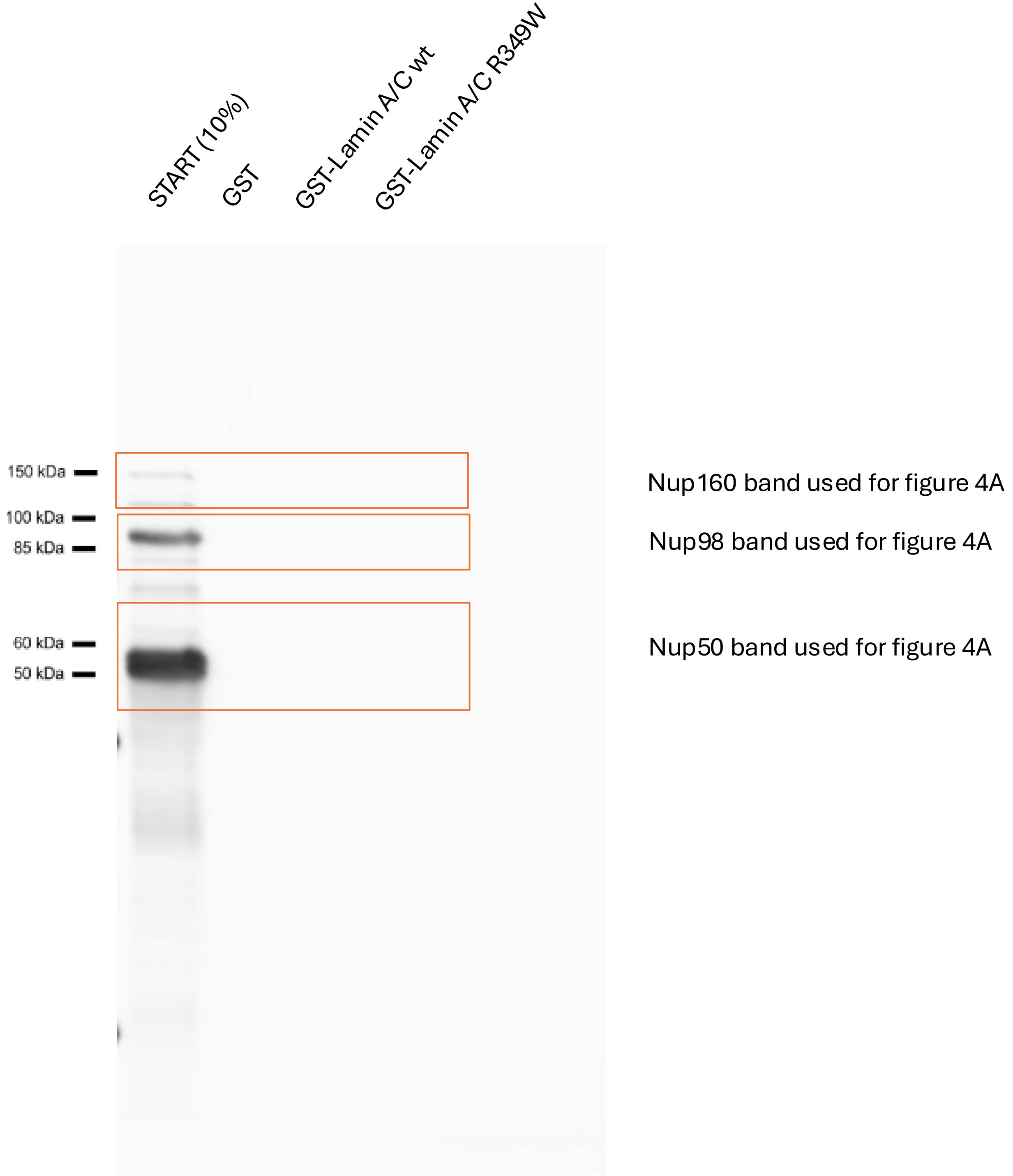
START (10%)
GST
GST-Lamin A/C wt
GST-Lamin A/C R349W
150 kDa
100 kDa
85 kDa
60 kDa
50 kDa
Nup160 band used for figure 4A
Nup98 band used for figure 4A
Nup50 band used for figure 4A

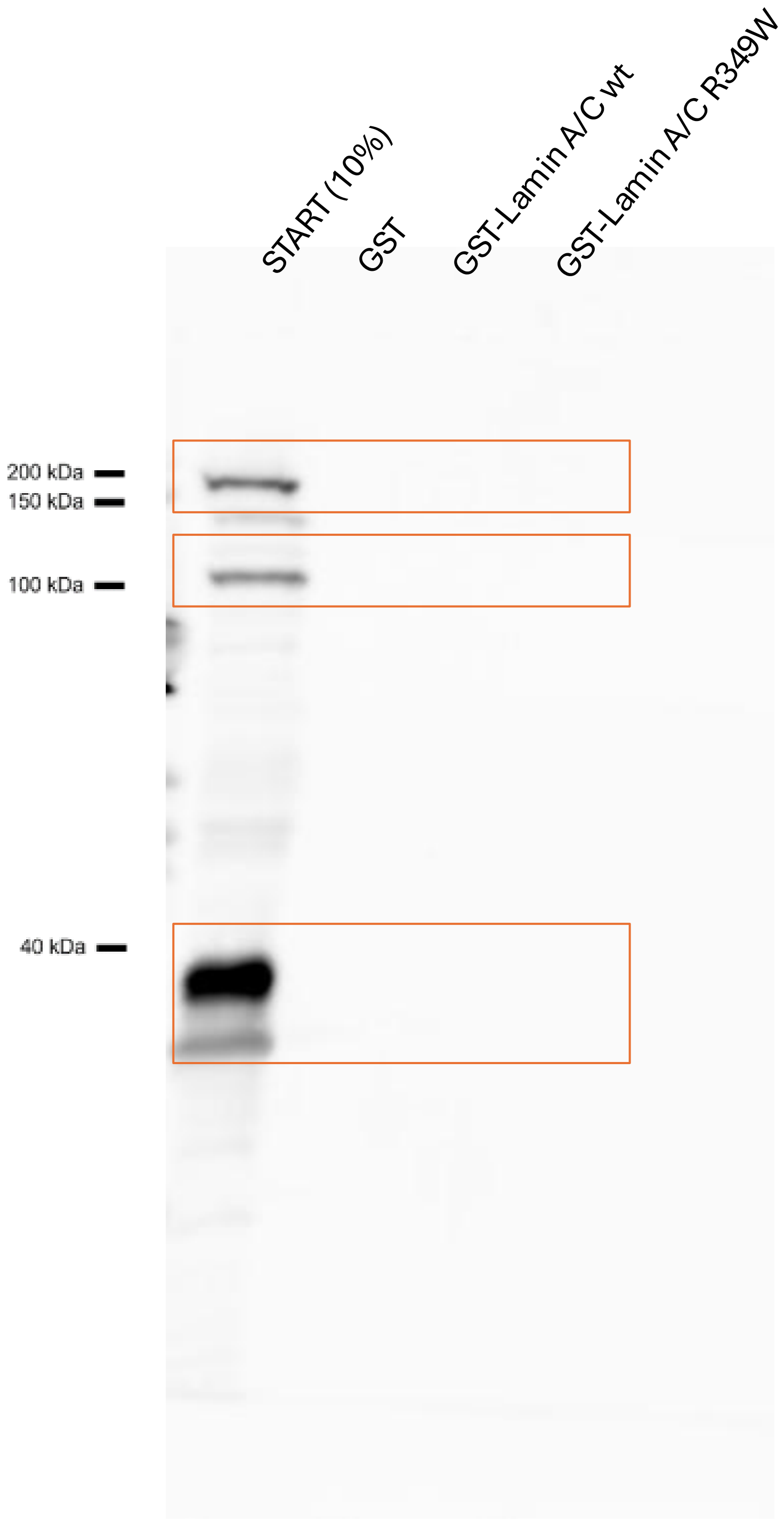

Nup188 band used for figure 4A

Nup107 band used for figure 4A

Nup53 band used for figure 4A

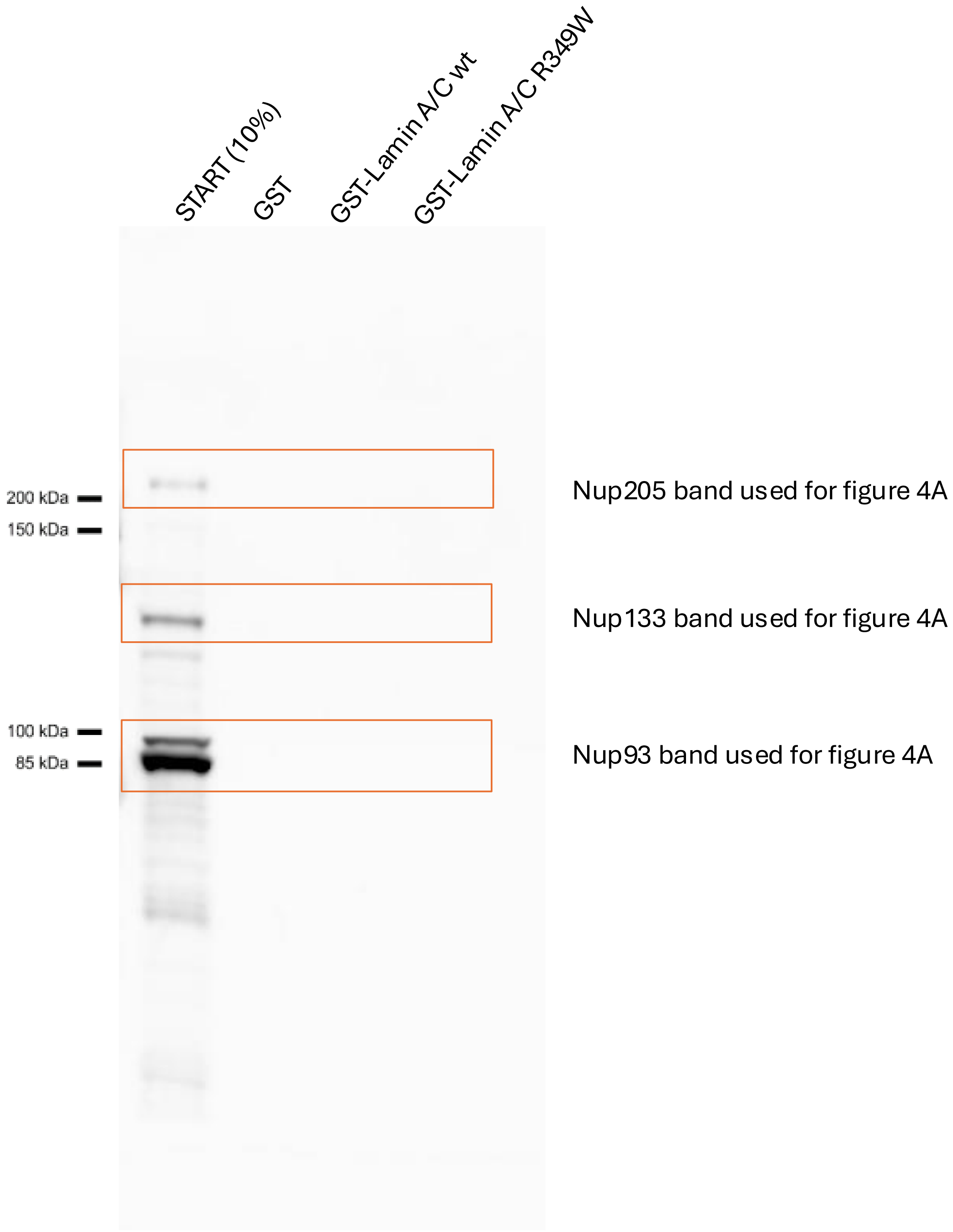
START (10%)
GST
GST-Lamin A/C wt
GST-Lamin A/C R349W
200 kDa
150 kDa
100 kDa
85 kDa
Nup205 band used for figure 4A
Nup133 band used for figure 4A
Nup93 band used for figure 4A
